# Multi-omics analysis of ST3GAL4-mediated lacto/neolacto glycosphingolipid metabolism reveals immune evasion and poor prognosis in TNBC

**DOI:** 10.3389/fimmu.2026.1760560

**Published:** 2026-04-22

**Authors:** Yu Zhang, Shuhan Wang, Xudong Yu, Kang Sun, Shengying Wang

**Affiliations:** Department of Thyroid Gland and Breast Surgery, The First Affiliated Hospital of Anhui University of Chinese Medicine, Anhui, Hefei, China

**Keywords:** glycosphingolipid metabolism, immune evasion, multi-omics, ST3GAL4, triple-negative breast cancer, tumor microenvironment

## Abstract

**Background:**

Triple-negative breast cancer (TNBC) is a highly aggressive subtype lacking effective targeted therapies. Increasing evidence highlights metabolic reprogramming as a hallmark of tumor progression and immune evasion. However, in this context, the specific metabolic–immune mechanisms underlying TNBC remain unclear.

**Methods:**

We performed an integrative multi-omics analysis combining bulk RNA-seq, single-cell RNA-seq, and spatial transcriptomics across TNBC and non-TNBC samples from TCGA, GEO, and 10X Genomics. Eighty-five KEGG metabolic pathways were profiled to identify TNBC-specific alterations. Machine learning models (Random Forest, XGBoost) were used to prioritize key metabolic genes. Immune infiltration was evaluated using CIBERSORT, ssGSEA, and ESTIMATE algorithms. Validation was conducted through immunohistochemistry (IHC) on 100 clinical samples from The First Affiliated Hospital of Anhui University Chinese Medicine.

**Results:**

The Lacto/Neolacto glycosphingolipid metabolism pathway was markedly activated in TNBC compared to adjacent and non-TNBC tissues, correlating with worse prognosis. Machine learning identified ST3GAL4 as the core enzyme within this pathway. High ST3GAL4 expression was associated with increased infiltration of regulatory T cells (Tregs) and M2 macrophages, reduced CD8^+^ T-cell activity, and enhanced epithelial–mesenchymal transition. Spatial transcriptomics confirmed localized enrichment of immunosuppressive cells in ST3GAL4-high regions. IHC validation demonstrated that ST3GAL4 overexpression in TNBC tissues predicts poor clinical outcomes.

**Conclusions:**

ST3GAL4-driven glycosphingolipid metabolism promotes tumor immune evasion and aggressiveness in TNBC. This metabolic–immune coupling axis represents a potential therapeutic target, offering mechanistic rationale for combining metabolic and immune checkpoint blockade strategies.

## Background

Breast cancer remains one of the leading causes of cancer-related morbidity and mortality among women worldwide (1, 2). With more than two million new diagnoses annually, breast cancer poses a significant global health burden, accounting for nearly 700,000 deaths per year (3). Among the various subtypes of breast cancer, triple-negative breast cancer (TNBC) is particularly challenging, as it lacks the expression of estrogen receptors (ER), progesterone receptors (PR), and human epidermal growth factor receptor 2 (HER2) (4). The absence of these receptors makes conventional targeted therapies ineffective, and TNBC’s inherent molecular heterogeneity, aggressive progression, and high rate of metastasis further complicate treatment outcomes (5, 6). Consequently, the prognosis for patients with TNBC remains poor, underscoring the need for novel therapeutic approaches (7, 8).

One of the hallmark features of cancer cells is metabolic reprogramming, which enables tumors to adapt to the heightened energy demands associated with rapid cell division (9). This altered metabolism not only provides energy but also facilitates other critical biological processes, including invasion, metastasis, and immune evasion (10). Tumors commonly rewire key metabolic pathways, such as glycolysis, fatty acid metabolism, and amino acid metabolism, to meet their proliferative needs (11). Notably, lactate metabolism, fatty acid synthesis, and amino acid catabolism have all been implicated in the aggressive progression of various cancer types (12). Targeting these reprogrammed metabolic pathways has emerged as a promising therapeutic strategy (13). Inhibitors targeting glucose metabolism and fatty acid metabolism have demonstrated potential as adjunctive treatments, highlighting the importance of metabolic pathways in cancer therapy.

Despite significant advancements in understanding the metabolic alterations in cancer, the specific metabolic characteristics of TNBC remain inadequately explored (14). Given the complexity of TNBC and its distinct molecular and metabolic landscape, identifying metabolic pathways that are uniquely altered in this subtype could provide valuable insights into potential therapeutic targets. Therefore, these findings suggest that this study aims to elucidate the metabolic features specific to TNBC and to explore their potential as novel targets for therapeutic intervention (15).

To strengthen the context and methodology of this study, recent advancements in the field of TNBC and metabolic reprogramming have been reviewed. Notably, studies published in the past three years have provided new insights into the molecular mechanisms of TNBC and its metabolic alterations. For instance, recent research (16) has highlighted the role of metabolic reprogramming in the progression of TNBC, particularly focusing on the dysregulation of glycolysis and fatty acid metabolism. Similarly, other studies (17, 18) have explored the immune microenvironment in TNBC, shedding light on how metabolic pathways influence immune evasion in this aggressive cancer subtype. Additionally, recent findings (19–21) have underscored the potential of targeting metabolic pathways as a therapeutic strategy in TNBC, reinforcing the relevance of our approach in identifying novel metabolic targets, such as ST3GAL4, that could enhance treatment outcomes. These recent publications contribute to the evolving understanding of TNBC, providing a robust foundation for the current study’s exploration of metabolic pathways and their role in immune infiltration.

This study investigates the unique metabolic features of TNBC and their interplay with the immune microenvironment. By analyzing 85 metabolic pathways from the KEGG database, we identified the Lacto/Neolacto glycosphingolipid metabolism pathway as significantly activated in TNBC. Using machine learning approaches, we further pinpointed ST3GAL4 as a key enzyme involved in this pathway. High expression of ST3GAL4 was found to be associated with increased infiltration of regulatory T cells (Tregs) and immune evasion. Immunohistochemical analysis from a cohort of breast cancer patients at The First Affiliated Hospital of Anhui University Chinese Medicine confirmed that ST3GAL4 expression is significantly higher in TNBC tissue and correlates with poor clinical outcomes.

## Methods

### Data acquisition

This study involved a large collection of transcriptomic, single-cell, and spatial transcriptomic data. For transcriptomic data, we integrated and analyzed gene expression data from 33 different cancer types along with their associated clinical information from The Cancer Genome Atlas (TCGA). Gene expression data were downloaded from the TCGA website in HTSeq-Counts or FPKM (Fragments Per Kilobase Million) format, covering a variety of tumor types. The downloaded data were standardized, including the removal of low-expressed genes, imputation of missing values, and normalization (RPKM conversion). Additionally, the GSE58812 (22) transcriptomic data and clinical information were processed and incorporated into the study. The GSE176078 (23) single-cell sequencing dataset, which includes 16 None TNBC 10 TNBC tissue samples, was also included. Spatial transcriptomic data were obtained from the 10X official website (https://www.10xgenomics.com/). The integration of these datasets provided comprehensive support for the in-depth analysis of tumor characteristics. The mechanism diagram is in **Supplementary Figure 1** of the manuscript. The flowchart for the selection process of TCGA breast cancer data is in **Supplementary Figure 2**.

### Metabolic pathways

We downloaded and curated gene information for 85 metabolic pathways from the KEGG database, covering critical biological processes such as fatty acid metabolism, RNA metabolism, carbohydrate metabolism, and amino acid metabolism. Each pathway includes detailed information about key genes and enzymes involved in relevant metabolic reactions. To ensure data accuracy and completeness, we rigorously screened and standardized these genes, providing a reliable foundation for subsequent functional analysis and biomarker exploration.

### GSVA scoring of metabolic pathways

We employed the GSVA method for the analysis of metabolic pathways, which is an unsupervised approach that evaluates the activity of a gene set in each sample by calculating the variation in gene expression within the set. Gene information for 85 metabolic pathways was downloaded and curated from the KEGG database to ensure the consistency and applicability of the gene sets in this study. For the analysis, we used the gsva() function from the GSVA package to calculate the gene set scores. All analyses were conducted in R 4.3.3, with GSVA package version 1.34.0. The computation was carried out using the ssGSEA method for scoring, with default parameters: min.sz = 5 (minimum gene set size) and max.sz = 500 (maximum gene set size).

### Enrichment analysis

Differentially expressed genes (DEGs) were subjected to functional annotation analysis. We used the clusterProfiler package for Gene Ontology (GO) biological process (BP), molecular function (MF), and cellular component (CC) enrichment analysis, as well as KEGG pathway enrichment analysis to uncover potential signaling pathways. The significance threshold was set at p.adjust < 0.05. Results were visualized using bubble plots and bar plots of enrichment.

### Single-cell data processing

A total of 26 breast cancer single-cell RNA sequencing (scRNA-seq) datasets were meticulously subjected to rigorous quality control (QC) and preprocessing to ensure the integrity and reliability of the data for downstream analyses. To remove low-quality cells, stringent QC thresholds were applied: cells with fewer than 200 detected genes (nFeature < 200), fewer than 500 total counts (nCount < 500), or mitochondrial gene expression exceeding 10% (percent.mt > 10%) were excluded from further analysis. These steps were implemented to minimize the inclusion of damaged or stressed cells, ensuring that only high-quality data were retained for analysis. Furthermore, doublets (cells that contain the RNA from more than one cell) were identified and excluded using the DoubletFinder method, with a probability threshold of 0.25 to classify potential doublets. This step is crucial as doublets can confound downstream analysis by introducing noise and artifacts, particularly in single-cell analyses.

Data normalization was performed using the SCTransform method in Seurat (version 4.0), which accounts for differences in sequencing depth between samples. This normalization process adjusts for technical biases, such as sequencing depth and gene expression variation, ensuring that differences in gene expression are reflective of biological variation rather than technical artifacts.

To mitigate batch effects and ensure that the datasets from different sources were comparable, Harmony (version 0.1.0) was employed for batch correction. Harmony is a widely used method that aligns datasets by adjusting principal components (PCs) that capture technical variations while preserving biological signals. This method was applied to the top 30 principal components, ensuring a high-quality, integrated dataset. To validate the effectiveness of batch effect removal, diagnostic plots, including PCA and t-SNE, were generated both before and after integration. These plots demonstrated the successful reduction of batch-related variability and confirmed that the integrated datasets reflected true biological differences across the samples.

For downstream analysis, genes with the highest variability across cells were selected using Seurat’s FindVariableFeatures function, which identified the top 2,000 most variable genes. These variable genes are considered the most informative for distinguishing different cell types and capturing biologically meaningful signals. Subsequently, data integration was performed using Seurat’s canonical correlation analysis (CCA)-based method, which allows for the combination of datasets from multiple sources while minimizing batch effects. This method is particularly useful when integrating scRNA-seq data from different experimental conditions or platforms. The top 30 principal components were selected for alignment to ensure accurate integration.

Cell types were annotated using a combination of automated methods and marker genes. Seurat’s SingleR function was applied, referencing the Human Primary Cell Atlas to identify and annotate cell types based on known gene expression profiles. Additionally, known breast cancer cell markers were used to further refine the cell type annotations, ensuring accurate categorization of the cells within the breast cancer context.

To investigate cellular differentiation and developmental trajectories, Monocle 2 was applied to infer pseudotime, an ordered representation of cell differentiation. The top 1,000 variable genes were used to build a trajectory, with cells ordered along pseudotime to track differentiation pathways. This method allowed for a better understanding of the cellular processes and developmental states within the tumor microenvironment.

For genomic analysis, Copy Number Variation (CNV) was assessed using inferCNV (version 1.6.0). CNV analysis is essential for identifying genomic alterations that are characteristic of malignant cells. A 100-gene window was used for CNV detection, and a high-confidence call threshold of 0.1 was applied. Normal cells were used as a reference for comparison, allowing the identification of copy number alterations associated with cancer cells.

Cell-to-cell communication within the tumor microenvironment was analyzed using CellChat (version 1.0.0) to identify ligand-receptor interactions between distinct cell populations. Interactions were initially filtered using a minimum communication strength threshold of 0.5, and robustness was confirmed by performing sensitivity analyses with alternative thresholds (0.4 and 0.6), which yielded consistent interaction patterns.

### Spatial transcriptomic data processing

We used the Seurat package to load spatial transcriptomic data from 10X Genomics (using the Load10X_Spatial function; https://www.10xgenomics.com/). Following quality control, we visualized the distributions of genes and transcripts. SCTransform was applied for normalization, and principal component analysis (PCA) was performed for dimensionality reduction. Adjacency graphs were constructed based on the principal components to facilitate clustering analysis. For spatial visualization, UMAP and SpatialPlot were used to display transcriptomic features across different regions.

Regarding the spatial resolution of the 10X Genomics Visium platform, we acknowledge that its resolution is approximately 55μm, which may not distinguish individual cells. In response to the concern about the colocalization of “ST3GAL4-high regions” with immunosuppressive cells, we first examined single-cell transcriptomic data. This analysis revealed that ST3GAL4 expression is predominantly enriched in epithelial cells, with negligible expression in other cell types. This indicates that the ST3GAL4 signal observed in the spatial transcriptomics data primarily reflects epithelial cell populations rather than a potential artifact arising from the spot size or mixed-cell signals.

To further validate the spatial association, we performed SCT deconvolution analysis, which confirmed a significant colocalization between ST3GAL4-high regions and immunosuppressive cells. These complementary analyses provide converging evidence to support the observed association, while also acknowledging the inherent limitations of the spot-based spatial resolution.

### Immune infiltration analysis

Normalized transcriptomic data were downloaded from TCGA, and CIBERSORT was used to compute the relative abundance of 22 immune cell subtypes. Samples with p-values > 0.05 were excluded to ensure result reliability. The ESTIMATE algorithm was used to assess immune scores, stromal scores, and tumor purity. To further quantify the activity of immune pathways, we used ssGSEA to analyze the activity of immune-related gene sets and comprehensively characterize the immune infiltration features of the tumor microenvironment.

### Machine learning

Feature modeling was performed using Random Forest (n_estimators=500, max_depth=6, min_samples_split=4, criterion=‘gini’) and XGBoost (learning_rate=0.05, max_depth=5, subsample=0.8, colsample_bytree=0.8). The prediction target in our study was based on clinical outcomes, such as relapse status or patient survival, with the target variable being binary (e.g., early relapse vs. late relapse). To ensure a robust evaluation of the model’s performance, we employed 5-fold cross-validation to split the dataset into training and validation sets. This approach helps reduce the risk of overfitting and ensures the model’s generalizability.

For feature selection, we used the Boruta algorithm for feature ranking, running it over 20 iterations to identify the most relevant features. Additionally, Recursive Feature Elimination (RFE) was applied to iteratively remove less informative features, further enhancing model performance. The model’s performance was assessed using ROC AUC scores, which were computed for each cross-validation fold, as well as the mean AUC score across all folds. This metric is particularly useful for imbalanced datasets, providing a reliable measure of model performance (**Supplementary Materials**).

To ensure the reproducibility of the analysis, we have included the complete code for data preprocessing, feature selection, model training, and evaluation in the **Supplementary Materials**. This will allow other researchers to replicate our results. Furthermore, we have provided detailed information about the specific libraries and their versions used in our analysis to ensure consistency and reliability when reproducing the workflow.

### Immunohistochemistry

This retrospective cohort study included 100 formalin-fixed, paraffin-embedded (FFPE) primary invasive breast carcinoma specimens resected at The First Affiliated Hospital of Anhui University Chinese Medicine between 2012 and 2024. The study protocol was approved by the institutional ethics committee, and all clinical data were de-identified prior to analysis. For each case, two board-certified pathologists selected representative tumor regions. Sections (4 µm thick) were cut, mounted on charged glass slides, and baked at 60 °C to promote tissue adhesion.

Prior to immunohistochemistry (IHC), slides were deparaffinized in xylene and rehydrated through graded ethanol. Antigen retrieval was performed in 10 mM citrate buffer (pH 6.0) by heat-mediated retrieval (microwave or pressure cooker) and allowed to cool to room temperature. Endogenous peroxidase activity was quenched with 3% hydrogen peroxide for 10 minutes, and non-specific binding was blocked with 5% normal serum or 5% bovine serum albumin (BSA) for 30 minutes at room temperature.

ST3GAL4 expression was detected using a validated primary antibody (anti-ST3GAL4). Antibody specificity was confirmed by orthogonal validation and by inclusion of tissue controls. The optimal working dilution (typically 1:50–1:200) was determined empirically. Primary antibody incubation was performed overnight at 4 °C, followed by incubation with a polymer-based HRP secondary reagent and visualization with diaminobenzidine (DAB). Slides were counterstained lightly with hematoxylin, dehydrated, cleared in xylene, and coverslipped.

Quality control measures included positive and negative control tissues in each staining batch. All reagent information, lot numbers, incubation times, buffer compositions, and instrument parameters were documented to ensure reproducibility.

For quantitative evaluation, digital image analysis was performed using ImageJ on high-resolution whole-slide images. Representative tumor regions were manually annotated by two independent pathologists blinded to clinical data. Color deconvolution was applied to separate the DAB signal, and standardized thresholds and cell-detection parameters were applied consistently across all samples. The average optical density (AOD) of DAB staining, reflecting the mean staining intensity within the annotated tumor area, was calculated automatically and used as the quantitative indicator of ST3GAL4 expression **Supplementary Table 1**.

All image analysis parameters were kept constant to minimize batch effects. The mean AOD value from two independent analyses was used for statistical evaluation.

### OS/DFS events

Overall Survival (OS) was defined as the time from diagnosis to death from any cause, while Disease-Free Survival (DFS) was defined as the time from diagnosis to the first occurrence of disease recurrence or metastasis. Both OS and DFS events were considered as failures when the event of interest (death or recurrence) occurred. In this study, the high/low groups for survival curves were defined based on the midpoint of the data.

### Statistical analysis

The Wilcoxon rank-sum test was used to assess differences between two groups. Prognostic analysis was conducted using the Kaplan-Meier method with the log-rank test. All statistical analyses were performed in R software (version 4.3.3, https://www.r-project.org/).

## Result

### Unique metabolic features of TNBC

**Figure 1:**
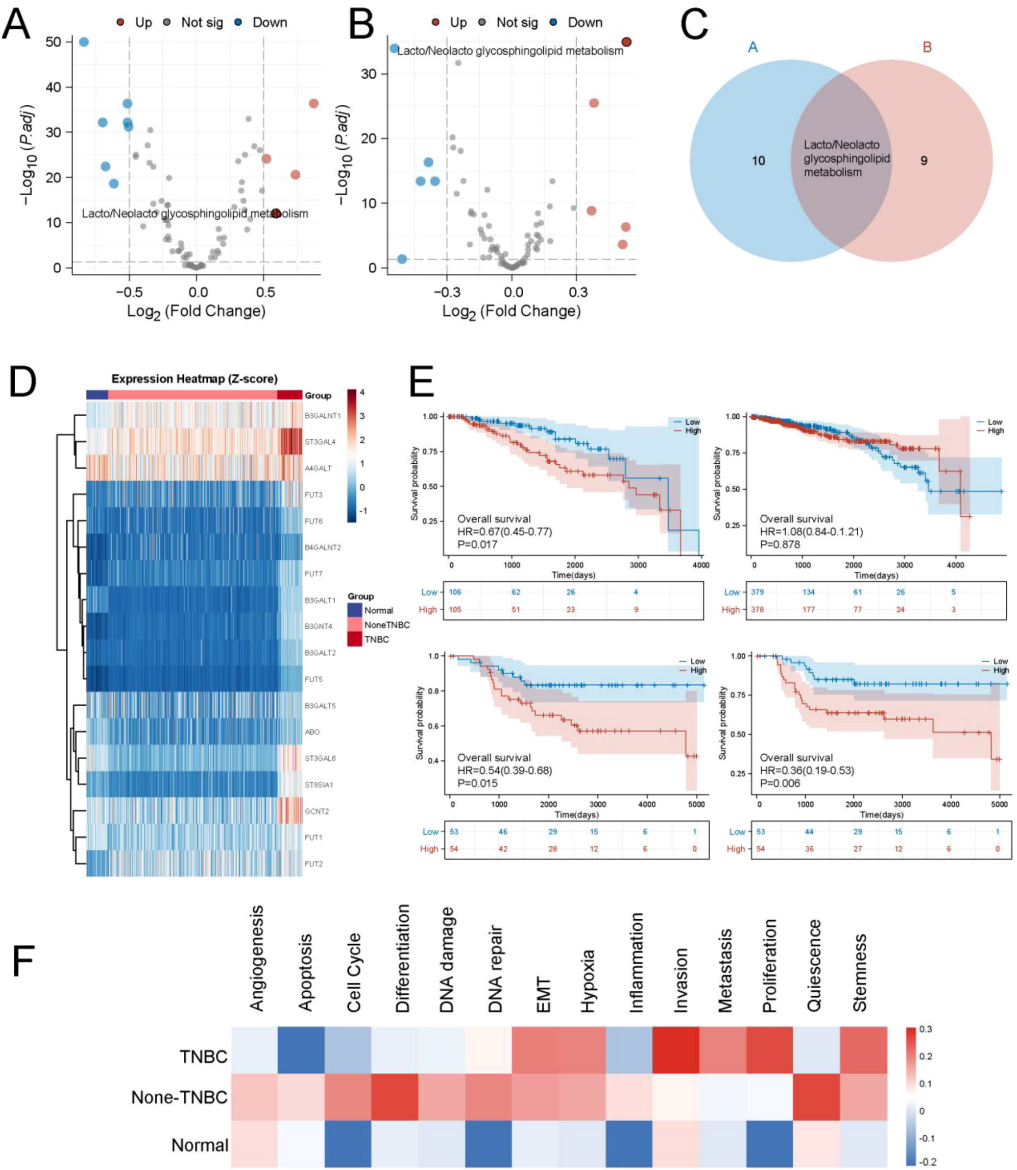
Lacto/Neolacto glycosphingolipid metabolism is a characteristic feature of TNBC patients. **(A, B)** Differential activity of 85 metabolic pathways between tumor and adjacent normal tissues, and between TNBC and non-TNBC patients. **(C)** Lacto/Neolacto glycosphingolipid metabolism was identified as the major overlapping pathway shared by both comparisons. **(D)** Expression levels of key genes involved in the Lacto/Neolacto glycosphingolipid metabolism pathway among adjacent normal tissues, non-TNBC, and TNBC tumors. **(E)** Prognostic impact of the Lacto/Neolacto glycosphingolipid metabolism score in TNBC patients based on TCGA and GSE58812 datasets. **(F)** Correlation analysis between the Lacto/Neolacto glycosphingolipid metabolism score and 13 tumor immune phenotypes.

We downloaded data from the KEGG database, which includes 85 common tumor-associated metabolic pathways, such as amino acid metabolism, carbohydrate metabolism, and DNA metabolism. To explore the unique metabolic features of TNBC, we compared the metabolic differences between TNBC and adjacent normal tissue, as well as between TNBC and non-TNBC. The results showed that, compared to adjacent tissues and non-TNBC, the activity of Lacto/Neolacto glycosphingolipid metabolism was significantly elevated in TNBC (**Figures 1A–C**). Further analysis of the expression of genes involved in this pathway revealed a marked increase in their expression in TNBC patients (**Figure 1D**). We then assessed the impact of Lacto/Neolacto glycosphingolipid metabolism on breast cancer prognosis (**Figure 1E**). Notably, in non-TNBC patients, the activity of this pathway was not significantly associated with prognosis. However, in this context, in TNBC patients, higher activity of this metabolic pathway correlated with poorer prognosis. This finding was validated in the TNBC cohort of the GSE58812 dataset, further supporting the association between elevated Lacto/Neolacto glycosphingolipid metabolism scores and poor prognosis. We further investigated the influence of this metabolic pathway on tumor behavior and found that, in TNBC, higher Lacto/Neolacto glycosphingolipid metabolism scores were strongly correlated with increased tumor invasiveness, metastasis, and epithelial-mesenchymal transition (EMT) (**Figure 1F**).

### ST3GAL4: a potential target for TNBC

**Figure 2:**
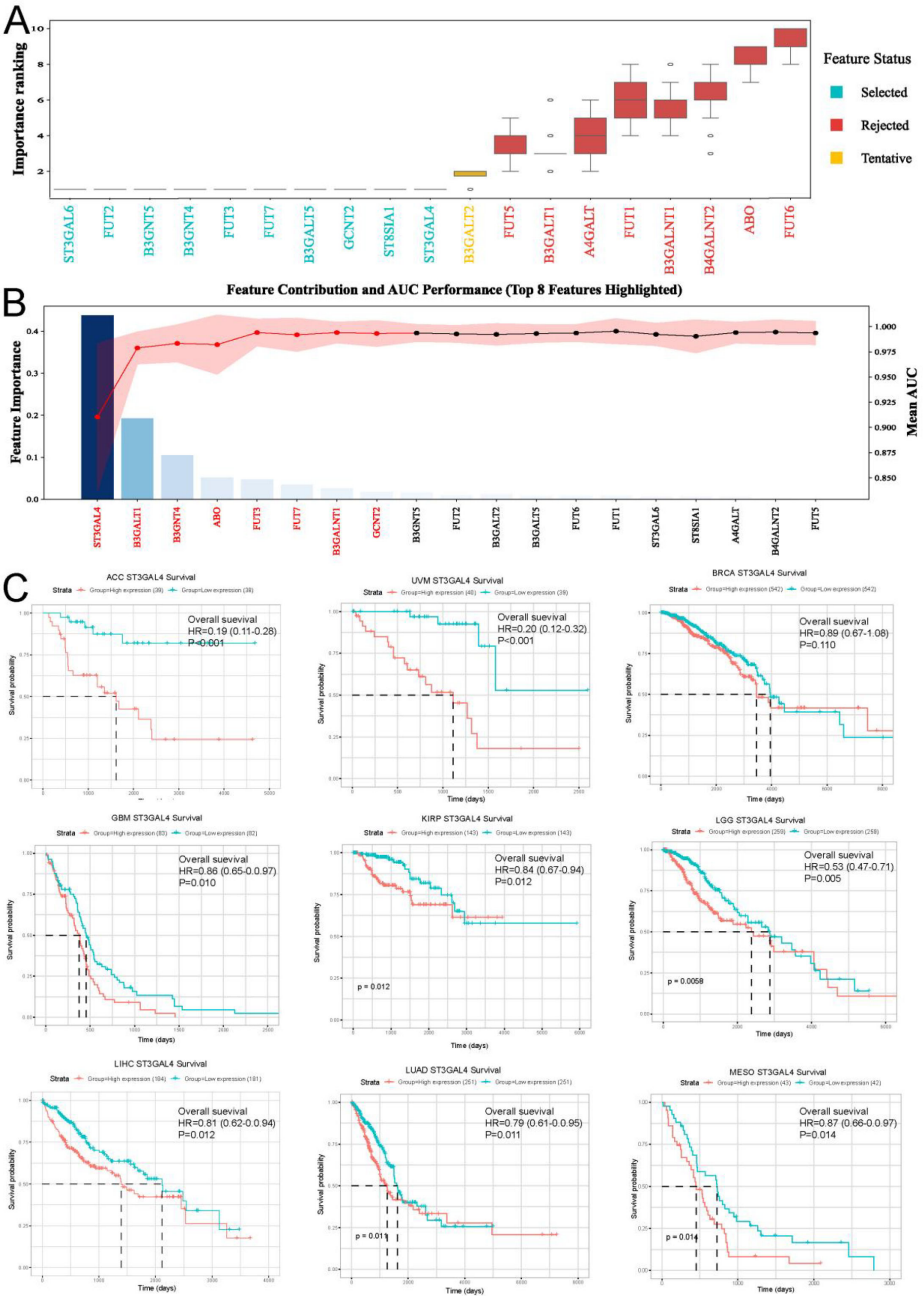
Machine learning highlights ST3GAL4 as a pivotal prognostic biomarker. **(A, B)** Machine learning–based feature selection identified ST3GAL4 as a key molecular determinant associated with poor prognosis in TNBC patients. **(C)** Pan-cancer survival analysis further confirmed that elevated ST3GAL4 expression is significantly linked to unfavorable outcomes in several tumor types, including ACC, UVM, GBM, and KIRP.

To identify key targets within the Lacto/Neolacto glycosphingolipid metabolism pathway, we employed machine learning algorithms to screen for potential critical genes. (**Supplementary Figure 3**). The results indicated that ST3GAL4 ranked highly in both algorithms and demonstrated excellent specificity for identifying TNBC (**Figures 2A, B**). We further validated the prognostic value of ST3GAL4 in a pan-cancer analysis. The findings revealed that in liver cancer, lung adenocarcinoma, and renal papillary carcinoma, higher expression of ST3GAL4 was associated with poorer prognosis (**Figure 2C**). Although ST3GAL4 is not a prognostic factor in overall breast cancer, higher expression levels of ST3GAL4 in TNBC patients were correlated with worse prognosis. The pan-cancer analysis of ST3GAL4 is in **Supplementary Figure 4**. The results of the univariate and multivariate analysis of ST3GAL4 combined with clinical variables are in **Supplementary Table 1**.

### Role of ST3GAL4 in the tumor immune microenvironment

**Figure 3:**
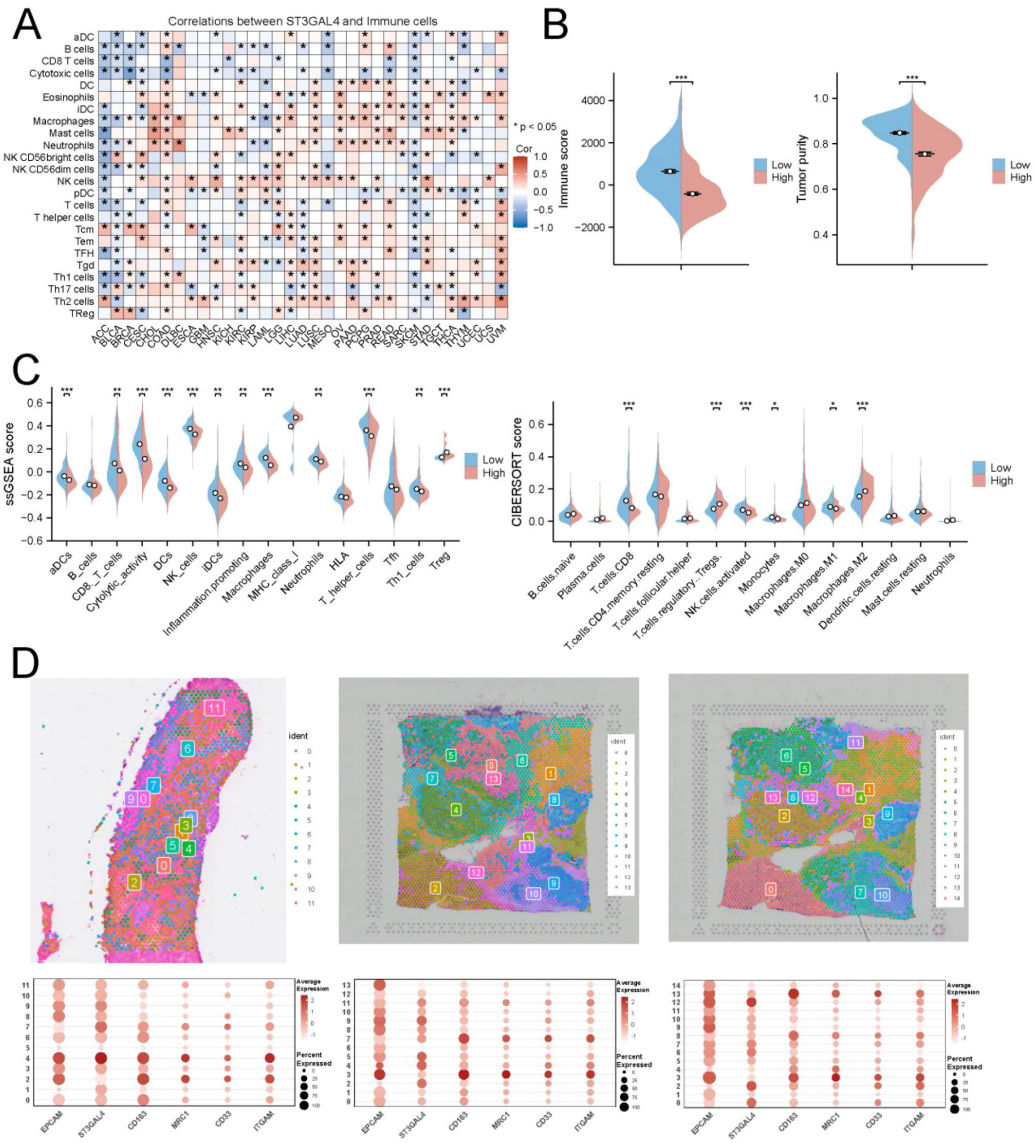
ST3GAL4 shapes an immunosuppressive tumor microenvironment in TNBC. **(A)** Pan-cancer correlation analysis revealed that ST3GAL4 expression was positively associated with immunosuppressive cell types, including regulatory T cells (Tregs), while showing strong negative correlations with CD8^+^ T cells and cytotoxic immune cells. **(B)** ESTIMATE analysis indicated that tumors with high ST3GAL4 expression exhibited significantly reduced immune scores and tumor purity compared with those with low ST3GAL4 expression. **(C)** Immune cell profiling by ssGSEA and CIBERSORT further demonstrated marked differences in immune infiltration patterns between the two expression groups. **(D)** Spatial transcriptomic analysis of breast cancer datasets from the 10x Genomics platform showed dense accumulation of immunosuppressive cells surrounding regions with elevated ST3GAL4 expression. * p<0.05, ** p<0.01, *** p<0.001.

To further investigate the role of ST3GAL4 in the tumor microenvironment, we conducted a pan-cancer analysis. Initial results suggested that ST3GAL4 was significantly negatively correlated with CD8+ T cells and cytotoxic T cells, while being positively correlated with immune-suppressive cells, such as Tregs (**Figure 3A**). Immune evaluation algorithms revealed that the tumor purity and immune scores were significantly lower in the ST3GAL4 high-expression group, indicating potentially poor responses to immunotherapy. Analysis using the CIBERSORT and ssGSEA algorithms further showed that the infiltration of immune-suppressive cells, such as Tregs and M2 macrophages, was significantly higher in the ST3GAL4 high-expression group, whereas the proportion of immune effector cells was significantly lower (**Figures 3B, C**). Spatial transcriptomic analysis further revealed that immune-suppressive cell infiltration was particularly prominent around regions of ST3GAL4 high expression (**Figure 3D**). Immune infiltration results obtained from additional computational algorithms are presented in **Supplementary Figure 5**.

### Unique expression of ST3GAL4 in malignant cells of TNBC

**Figure 4:**
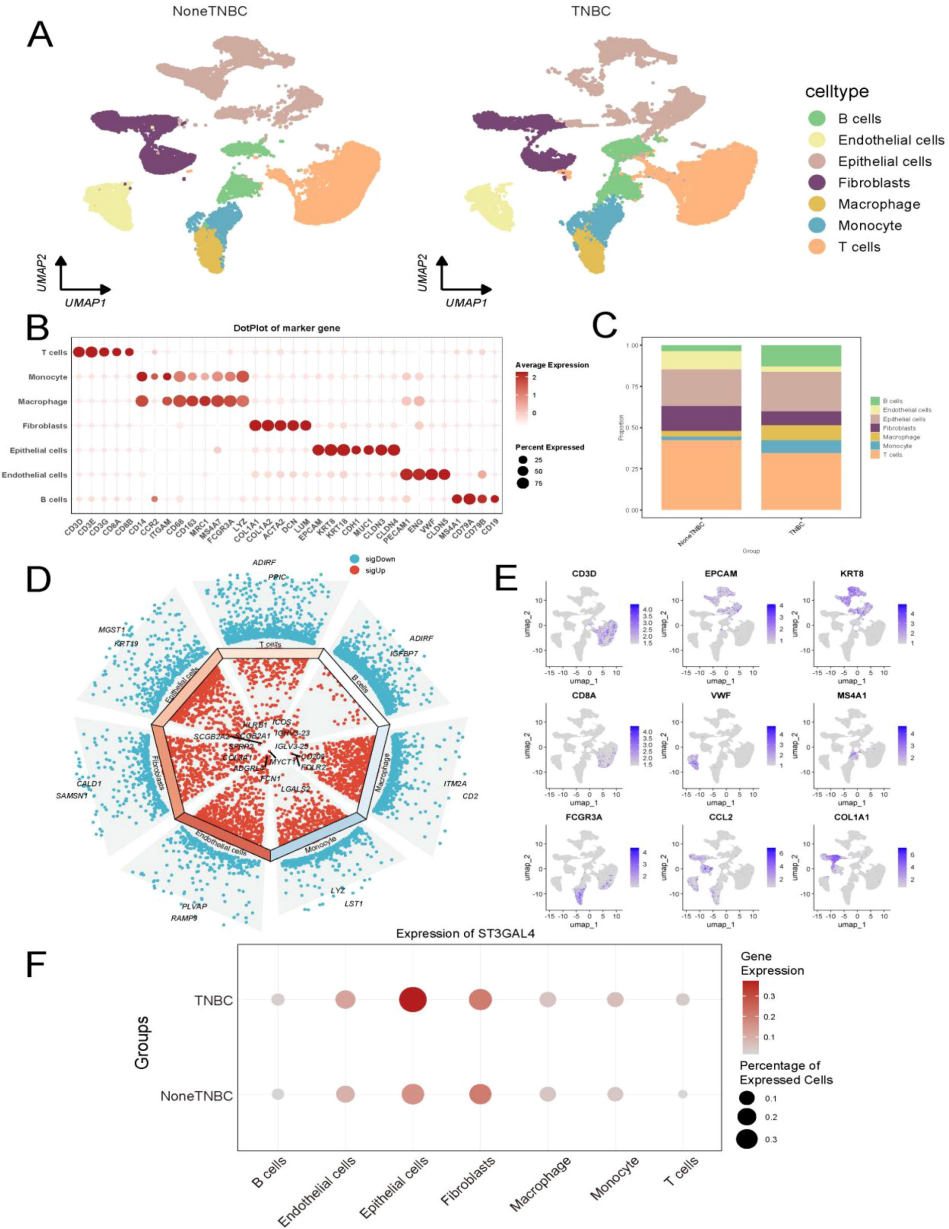
Single-cell analysis of GSE176078. **(A)** Cell-type annotation of clusters based on high-quality reference studies. **(B)** Annotation of clusters according to marker gene expression. **(C)** Proportional distribution of cell types between the two groups. **(D, E)** Expression patterns of selected genes across different cell types. **(F)** ST3GAL4 is predominantly expressed in malignant cells and shows the highest expression in TNBC.

We collected single-cell RNA-seq data from a cohort of 26 patients and performed quality control, ultimately including 88,147 cells for analysis (**Figures 4A, B, D, E**). Single-cell quality control results are presented in **Supplementary Figures 6**, **7**. The results indicated that in TNBC, the proportions of stromal cells and macrophages were significantly higher compared to non-TNBC (**Figure 4C**). Further investigation of ST3GAL4 expression revealed an unexpected finding: ST3GAL4 was primarily expressed in malignant cells, with the highest expression observed in TNBC (**Figure 4F**).

### High expression of ST3GAL4 promotes immune-suppressive cell infiltration

**Figure 5:**
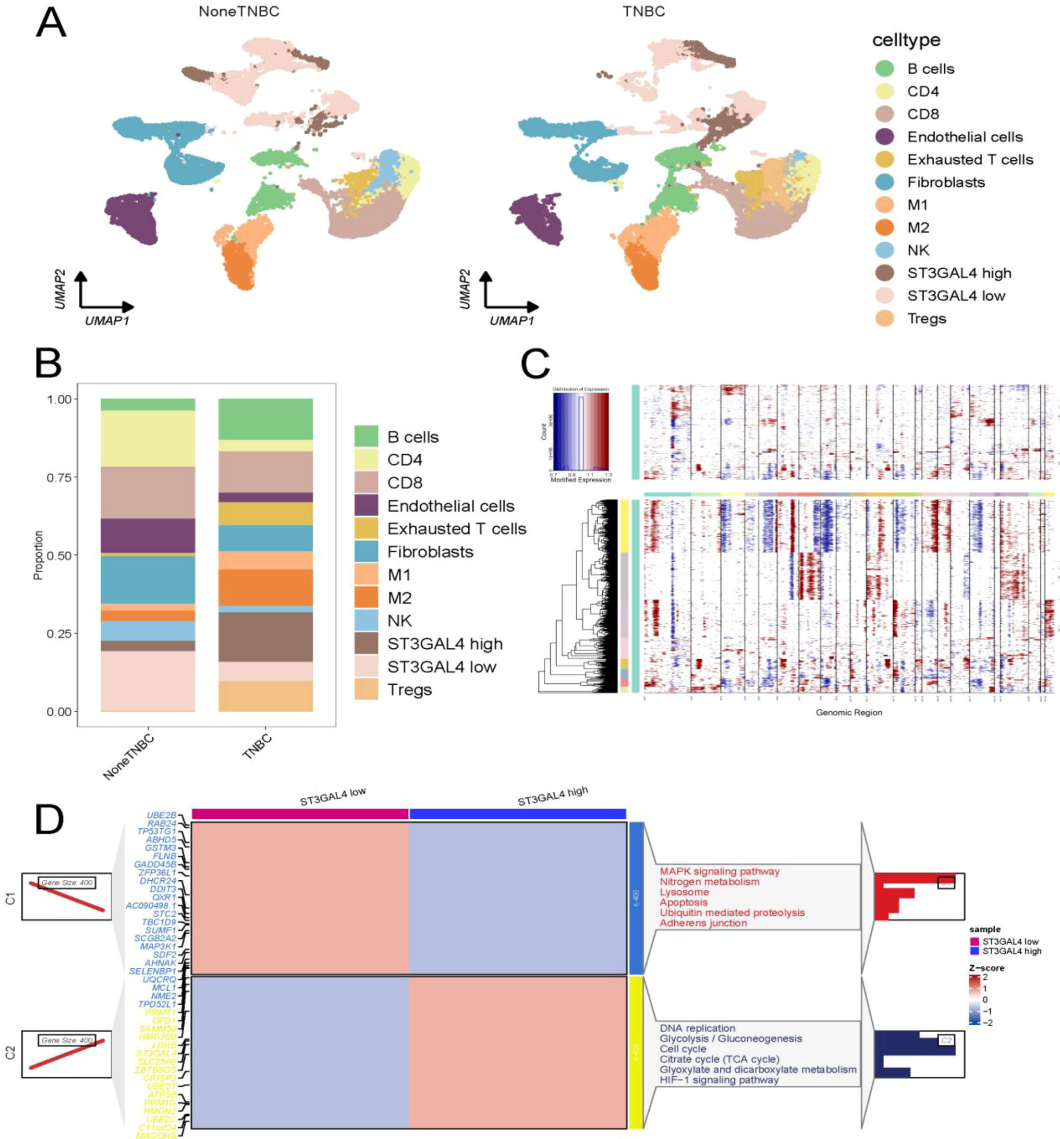
ST3GAL4 shapes the immunosuppressive landscape and genomic features of TNBC. **(A)** TNBC samples with high ST3GAL4 expression were predominantly associated with a more immunosuppressive tumor microenvironment. **(B)** The distribution of immune cell infiltration differed markedly between the high- and low-ST3GAL4 groups. **(C)** Tumors with elevated ST3GAL4 levels exhibited increased copy number amplifications and deletions, suggesting greater genomic instability. **(D)** Differential and enrichment analyses indicated that ST3GAL4-high cells were characterized by upregulated pathways involved in DNA damage repair, cell cycle regulation, glycolysis, and HIF signaling.

Based on the expression profile of ST3GAL4, we divided malignant cells into high and low expression groups. In non-TNBC, ST3GAL4 expression was higher, and the proportion of Tregs and M2 macrophages was also elevated, which is consistent with our previous transcriptomic data (**Figures 5A, B**). InferCNV analysis revealed that the ST3GAL4 high-expression group exhibited more genomic amplifications and deletions, indicating greater tumor cell heterogeneity (**Figure 5C**). Differential expression and enrichment analyses further demonstrated that the tumor cells in the ST3GAL4 high-expression group were enriched for functions related to DNA replication, cell cycle, TCA cycle, and HIF signaling pathways (**Figure 5D**).

### Single-cell analysis reveals the value of ST3GAL4 in the tumor microenvironment of TNBC

**Figure 6:**
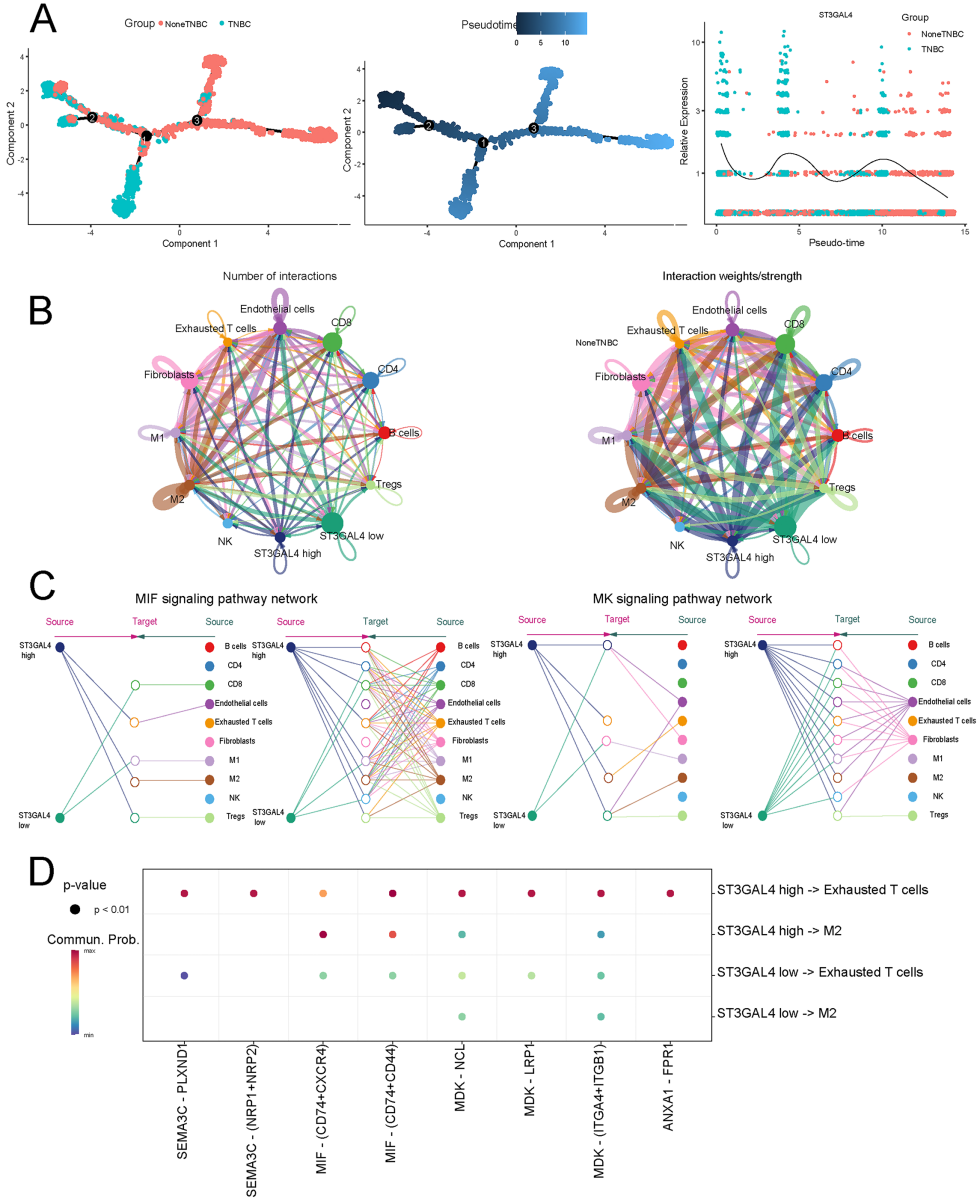
High ST3GAL4 expression mediates intercellular communication with immunosuppressive cells. **(A)** Pseudotime trajectory analysis showed that cells with elevated ST3GAL4 expression were positioned at more advanced differentiation stages. **(B)** The strength and number of intercellular communications were markedly increased in the ST3GAL4-high group compared with the ST3GAL4-low group. **(C)** ST3GAL4 mediated signaling interactions with immunosuppressive cells primarily through the MIF and MK pathways. **(D)** Predicted ligand–receptor pairs illustrate the potential communication network between ST3GAL4-expressing tumor cells and immunosuppressive immune cells.

We further utilized the Monocle2 algorithm to analyze the differentiation trajectories of cancer cells. The results showed that the expression of ST3GAL4 progressively increased as cells differentiated (**Figure 6A**). Tumor microenvironmental cell communication analysis revealed that malignant cells with high ST3GAL4 expression exhibited significantly higher frequency and intensity of interactions with immune-suppressive cells, particularly within the MK and MIF signaling pathways (**Figures 6B, C**). Moreover, analysis of receptor-ligand interactions showed that the interactions between ST3GAL4 high-expressing malignant cells and Tregs, as well as M2 macrophages, were significantly stronger compared to the ST3GAL4 low-expression group (**Figure 6D**).

### Validation in the first affiliated hospital of Anhui university Chinese medicine breast cancer cohort

**Figure 7:**
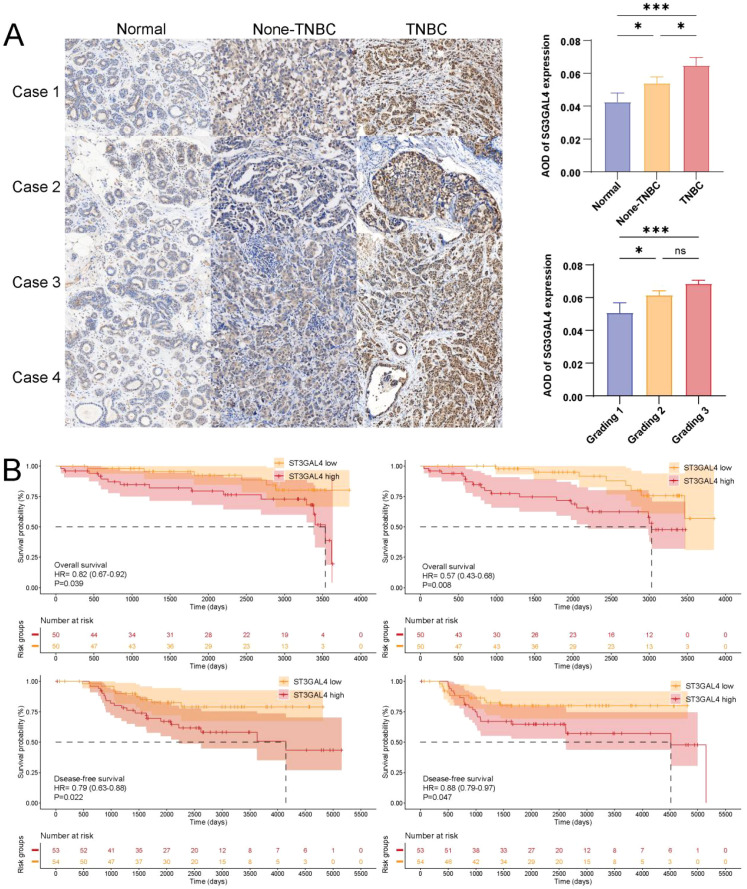
Immunohistochemical validation of ST3GAL4 expression in breast cancer tissues. **(A)** Analysis of more than 100 breast cancer specimens from The First Affiliated Hospital of Anhui University Chinese Medicine revealed a stepwise increase in ST3GAL4 protein expression from adjacent normal tissues to non-TNBC and TNBC samples, with expression positively correlated with tumor grade. **(B)** Kaplan–Meier survival analysis demonstrated that patients with high ST3GAL4 expression had significantly worse overall survival (OS) and disease-free survival (DFS) outcomes. * p<0.05, *** p<0.001.

Both mRNA and protein levels were consistently higher in tumor tissues than in adjacent normal tissues across all four TNBC patients (**Supplementary Figure 7**). We collected tissue samples and follow-up data from breast cancer patients at The First Affiliated Hospital of Anhui University Chinese Medicine and performed immunohistochemical staining to detect the expression of ST3GAL4 (**Figure 7A**). The baseline characteristics of the patients are provided in **Supplementary Table 2**. The results showed that in TNBC patients, ST3GAL4 expression was significantly higher compared to non-tumor tissues and non-TNBC patients. The expression of ST3GAL4 progressively increases with the progression of TNBC pathological grade. Further analysis revealed that high expression of ST3GAL4 was associated with poor prognosis. These findings suggest that ST3GAL4 could be a potential biomarker for TNBC and is closely linked to adverse prognosis, indicating its potential role in the pathophysiology of TNBC (**Figure 7B**). The results of univariate and multivariate Cox regression analyses are presented in **Supplementary Table 3**.

## Discussion

TNBC is one of the most aggressive subtypes of breast cancer, characterized by high molecular heterogeneity, strong invasiveness, and a lack of effective targeted therapies (24). In recent years, multi-omics studies have revealed that TNBC not only has unique transcriptional and signaling pathway characteristics but also exhibits significant metabolic reprogramming (25). Metabolic abnormalities have been recognized as important mechanisms driving TNBC progression, immune evasion, and treatment resistance (26). This study integrates transcriptomic, single-cell, and spatial transcriptomic data to systematically reveal the aberrant activation of the Lacto/Neolacto glycosphingolipid metabolism pathway in TNBC and identifies the key enzyme ST3GAL4 in tumor immune suppression formation. This provides a new biological perspective for understanding the metabolic-immune coupling mechanisms in TNBC.

Metabolic reprogramming is considered a “hallmark” feature of cancer cells that grants them a proliferation and survival advantage (27). Compared with hormone receptor-positive breast cancer, TNBC relies more on energy supply pathways such as glycolysis, fatty acid synthesis, and amino acid metabolism (28). However, in this context, beyond energy metabolism, the role of Lacto/Neolacto glycosphingolipid metabolism has recently garnered widespread attention (29). Glycosphingolipids are key components of the cell membrane, involved in cell signaling, adhesion, and immune recognition, and their metabolic disturbances have been closely linked to the invasiveness of breast cancer (30, 31).

Existing studies have shown that key enzymes in the glycosphingolipid metabolism pathway, such as B4GALNT1 and ST3GAL5, can regulate the glycan structure of tumor cell membranes, influencing the activity of EGFR, TGF-β, and PI3K/Akt signaling, thereby promoting EMT and metastatic potential (32, 33). This study found that the Lacto/Neolacto branch is specifically activated in TNBC, suggesting that TNBC may promote receptor clustering and sustained signal activation through glycan modifications. This aligns with the role of glycosylation as a “signal amplifier” in cancer biology (31, 34). In contrast to traditional studies focusing on energy metabolism, this study emphasizes glycan metabolism as a new regulatory hub in the TNBC metabolic network, with potential translational significance.

ST3GAL4 is a β-galactoside sialyltransferase responsible for catalyzing the sialylation of the sugar chain’s terminal (35). Sialylated glycans not only affect cell adhesion and signal transduction but also play a core role in immune evasion. Previous studies have shown that ST3GAL4 is highly expressed in various tumors and is closely associated with metastasis and immune suppression. For example, Munkley et al. reported that ST3GAL4 promotes cancer cell hematogenous dissemination by regulating E-selectin-dependent metastasis (36), while Yu et al. found that sialylation mediated by ST3GAL4 enhances the stability of PD-L1, thereby inhibiting T cell activation (37, 38).

This study further reveals that in TNBC, high ST3GAL4 expression is associated with tumor cell genomic instability and EMT activation, as well as a significant positive correlation with Treg cells and M2 macrophages. Combined with spatial transcriptomic results, it is observed that areas of high ST3GAL4 expression are enriched with immune-suppressive cell populations, suggesting that it may alter the tumor immune microenvironment through glycan modifications (39, 40). Notably, the immune evasion mechanism associated with ST3GAL4 may not solely depend on PD-L1 but rather involve complex receptor-ligand interactions mediated by various sialylated glycan modifications, such as Siglec family receptors recognizing sialylated glycans (41–43). This is highly consistent with the recent concept of a “glyco-immune checkpoint”.

Abnormal glycosphingolipid metabolism provides a new therapeutic entry point for TNBC. Experimental drugs, such as miglustat and genistein, which inhibit glycosphingolipid synthesis, have been shown to reduce cell migration in breast cancer models (44). Additionally, strategies targeting sialylation, such as sialidase-conjugated antibodies (E-Sel-sialidase), have been shown in animal experiments to enhance the efficacy of immune checkpoint inhibitors (ICIs) (45). Our study suggests that ST3GAL4 may be one of the most specific and clinically actionable targets in the glycosphingolipid metabolism pathway of TNBC, with high expression serving as a prognostic marker and potentially as a molecular target for metabolic-immune combination therapy.

It is important to emphasize that the immune response to TNBC treatment varies greatly, and the metabolic state may be a key determinant (46). If ST3GAL4-driven glycan remodeling can be effectively inhibited, it may reduce immune-suppressive infiltration and improve the response to immune therapies (47). Therefore, these findings suggest that future studies may explore combination strategies involving ST3GAL4 inhibitors and PD-1/PD-L1 antibodies or metabolic intervention drugs to achieve precision therapy (48, 49).

Despite the strong credibility provided by the multi-omics integration and clinical validation in this study, several areas for improvement remain. First, the precise molecular mechanisms by which ST3GAL4 mediates immune evasion require further investigation through functional glycomics and immune co-culture experiments. Second, as a retrospective study, the sample size and population composition may limit the statistical power and generalizability of the findings. Third, the dynamic metabolic-immune interactions in TNBC, which vary temporally and spatially, highlight the need for future studies that integrate spatial multi-omics techniques with metabolic flux analysis. These approaches will be essential for capturing the full complexity of the tumor microenvironment. Furthermore, while the current study primarily relies on bioinformatics mining and IHC validation of clinical samples, which provides valuable correlational insights, it is recommended to complement this with PCR or Western blotting (WB) for further validation of key findings. In addition, *in vitro* and *in vivo* functional experiments on the identified key genes are crucial to confirm their roles in TNBC. These additional efforts will further substantiate the functional and pharmacological potential of ST3GAL4 as a therapeutic target for metabolic-immune coupling in TNBC.

## Conclusion

In summary, using multi-omics analyses, this study systematically characterizes the aberrant activation of the Lacto/Neolacto glycosphingolipid metabolism pathway in TNBC and identifies ST3GAL4 as a key molecule associated with metabolic reprogramming and immunosuppressive microenvironments. This finding not only enriches the theoretical system of TNBC metabolic biology but also provides a solid foundation for the development of novel metabolic-immune combination therapies. Future studies focused on the functional validation and targeted intervention of ST3GAL4 may promote a shift in TNBC treatment strategies from single immune suppression to a new paradigm of metabolic regulation synergizing immune activation.

## Data Availability

Raw transcriptomic datasets were obtained from TCGA (https://www.cancer.gov/ccg/research/genome-sequencing/tcga), GEO (https://www.ncbi.nlm.nih.gov/geo/), and HRA (https://www.10xgenomics.com/). The results of the data analysis can be accessed at https://data.mendeley.com/datasets/k33n85s443/1.
