# Supplementary material for: Multi-omics analysis of ST3GAL4-mediated lacto/neolacto glycosphingolipid metabolism reveals immune evasion and poor prognosis in TNBC
Source: Front Immunol. 2026 Apr 22;17:1760560. doi: 10.3389/fimmu.2026.1760560 (PMC13143995; doi:10.3389/fimmu.2026.1760560)
Supplement: Supplementary file 1 [file DataSheet1.zip › Supplementary Materials/Ethical Approval Document.pdf]

# 安徽中医药大学第一附属医院医学伦理委员会
## 伦理审查批件

| | |
|---|---|
| 伦理审查批件号 | 2025AH-114 |
| 主要研究者 | 张雨 |
| 项目名称 | 复元活血汤在乳腺癌围手术期应用临床价值 |
| 资助来源 | 甲状腺乳腺外科 |
| 研究单位 | 安徽中医药大学第一附属医院 |
| 审查地点 | 合肥梅山路117号安徽中医药大学第一附属医院 |
| 审查类别/审查方式 | 初始审查申请/快速审查 |
| 审查时间 | 2025-07-24 |
| 审查委员 | 张皖东、张娟 |
| 审查文件 | 1. 初始审查申请表<br>2. 临床研究方案，版本号/日期：V1.0/2025年7月10日<br>3. 豁免知情同意申请表，版本号/日期：V1.0/2025年7月10日<br>4. 主要研究者专业履历及研究人员名单、职责分工<br>5. 回顾性调查表<br>6. 生物样本/信息数据来源证明<br>7. 立项证明 |
| 审查意见 | 根据国家卫生健康委等：关于印发涉及人的生命科学和医学研究伦理审查办法的通知国卫科教发（2023年），国家药监局，国家卫生健康委发布的“药物临床试验质量管理规范”（2020年），“医疗器械临床试验质量管理规范”（2022年），“药物临床试验伦理审查工作指导原则”（2010年），原卫计委“涉及人的生物医学研究伦理审查办法”(2016年)，国家中医药管理局“中医药临床研究伦理审查管理规范”（2010年），以及世界医学会《赫尔辛基宣言》（2024）等，经本伦理委员会审查，按所同意的临床研究方案、知情同意书开展本项研究。 |
| 伦理委员会声明 | 请遵循GCP原则、遵循伦理委员会批准的方案开展临床研究，保护受试者的健康与权利。<br>研究开始前，请申请人尽可能完成临床试验注册。 |

<table>
<tr><td></td><td colspan="3">研究过程中若变更主要研究者，对临床研究方案、知情同意书、招募材料等的任何修改，请申请人提交修正案审查申请。<br>请按照伦理委员会规定的年度/定期跟踪审查频率，审查人在截止日期前 1 个月提交研究进展报告；申办者应当向组长单位伦理委员会提交各中心研究进展的汇总报告；当出现任何可能显著影响试验进行、或增加受试者危险的情况时，请申请人及时向伦理会员会提交书面报告。<br>研究纳入了不符合纳入标准或符合排除标准的受试者，符合中止试验规定而未让受试者退出研究，给予错误治疗或剂量，给予方案禁止的合并用药等没有遵从方案开展研究的情况；或可能对受试者的权益/健康以及研究的科学性造成不良影响等违背 GCP 原则的情况，请申办者/监察员/研究者提交违背方案报告。<br>申请人暂停或提前终止临床研究，请及时提交暂停/终止研究报告。完成临床研究，请提交结题报告。</td></tr>
<tr><td>有效期</td><td>24 个月（2025-07-24 至 2027-07-24）</td><td>年度/定期跟踪审查频率</td><td>12 个月</td></tr>
<tr><td colspan="2">伦理委员会联系人及联系电话</td><td colspan="2">伦理办公室：0551-62838532</td></tr>
<tr><td colspan="2">伦理委员会主任/副主任委员签字及日期</td><td colspan="2">[signature] 2025-07-24</td></tr>
<tr><td colspan="4">安徽中医药大学第一附属医院（安徽省中医院）医学伦理委员会（盖章）</td></tr>
</table>
