# Supplementary figures and images for: Multi-omics analysis of ST3GAL4-mediated lacto/neolacto glycosphingolipid metabolism reveals immune evasion and poor prognosis in TNBC
Source: Front Immunol. 2026 Apr 22;17:1760560. doi: 10.3389/fimmu.2026.1760560 (PMC13143995; doi:10.3389/fimmu.2026.1760560)

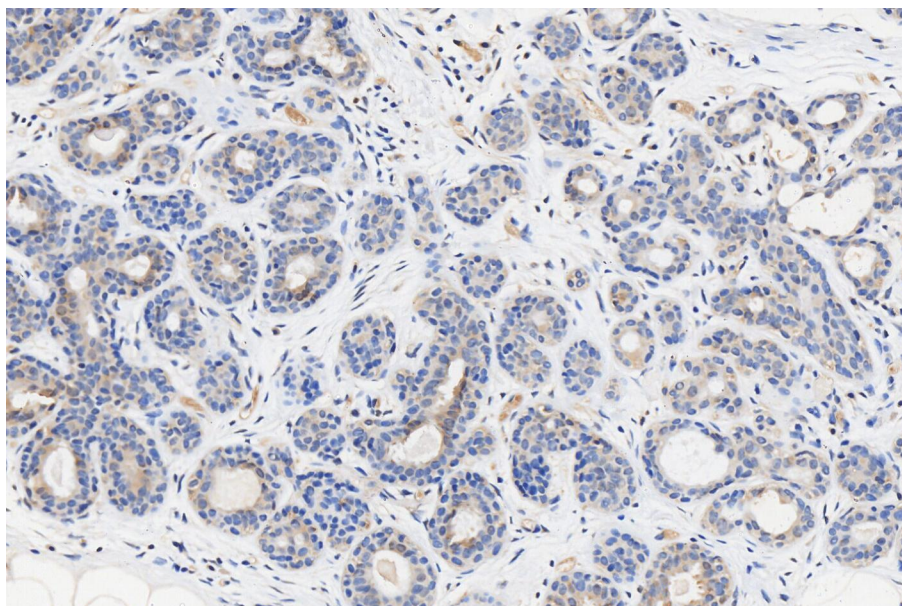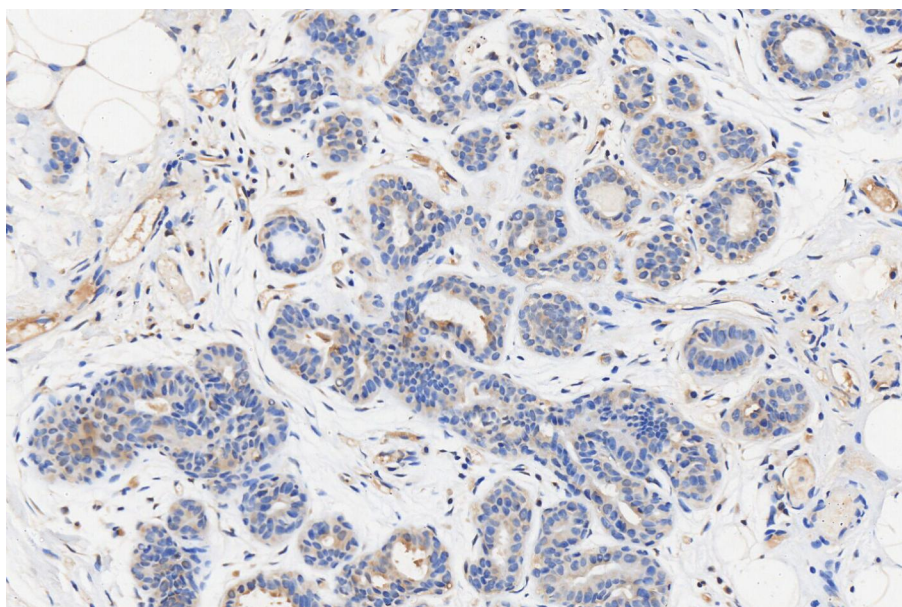

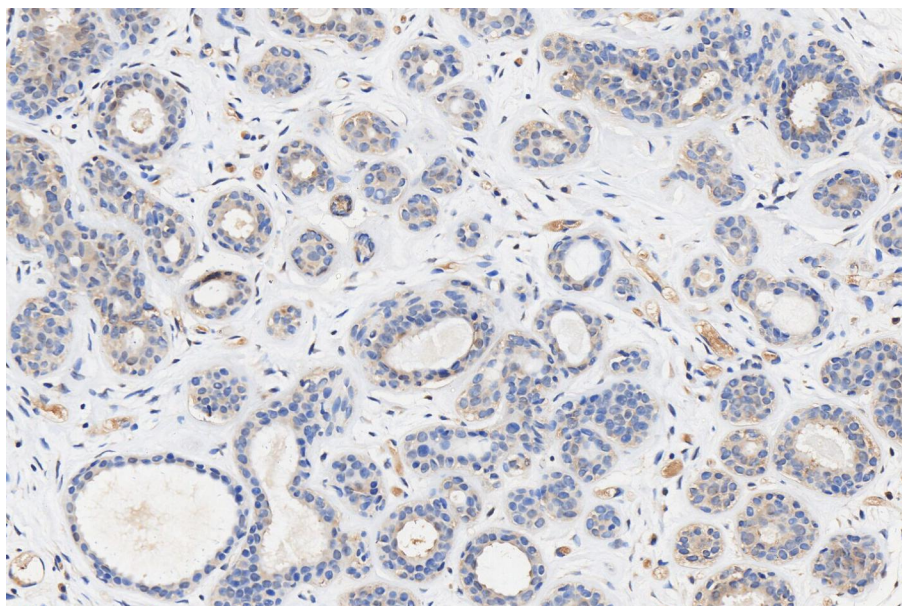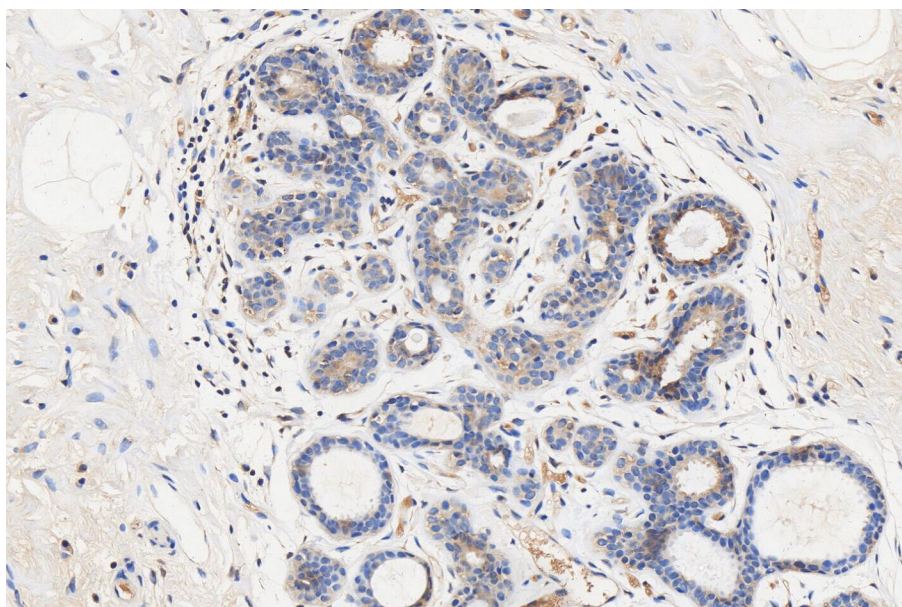

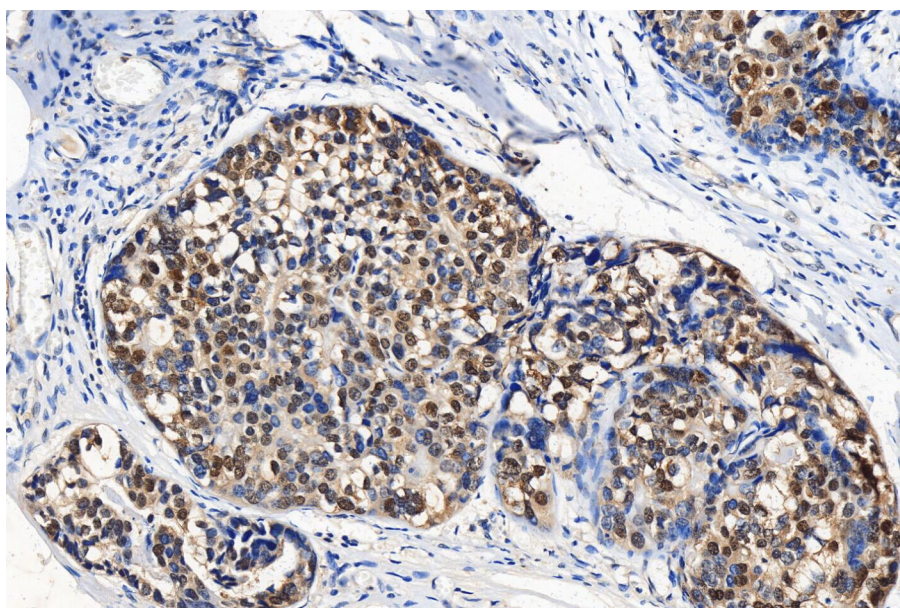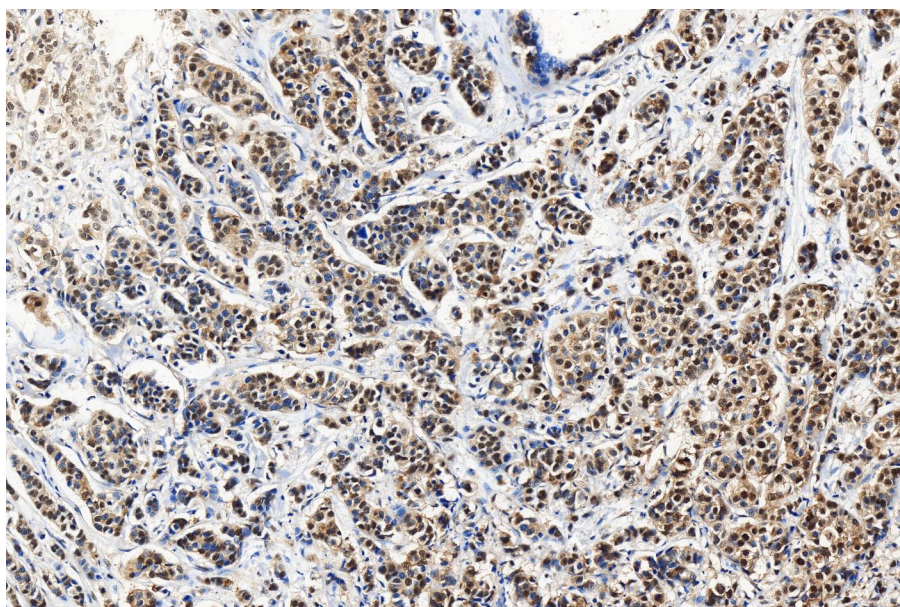

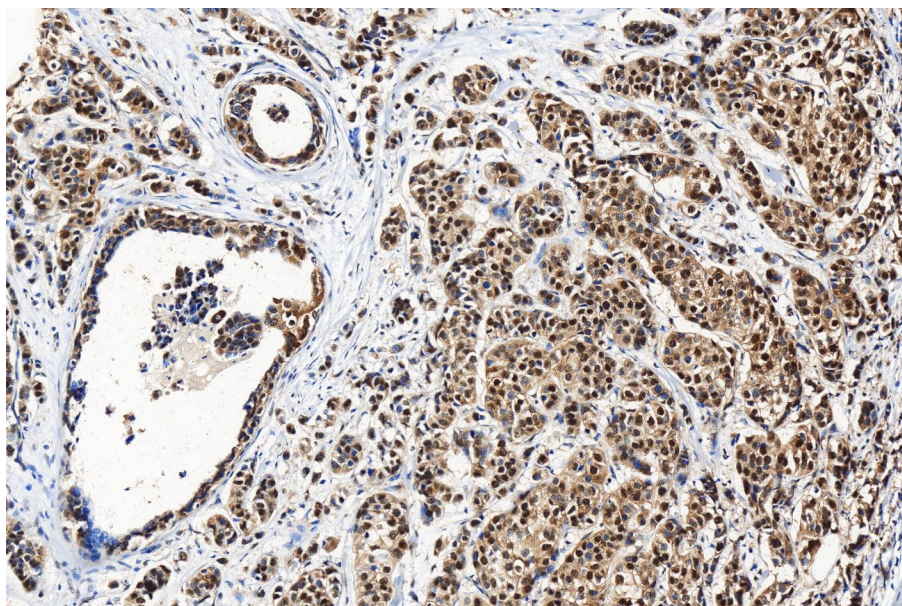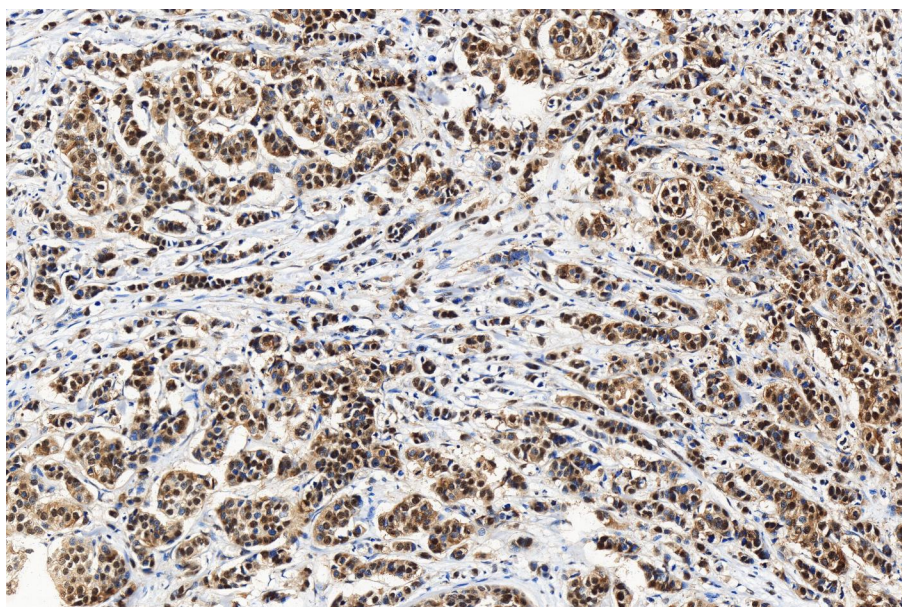

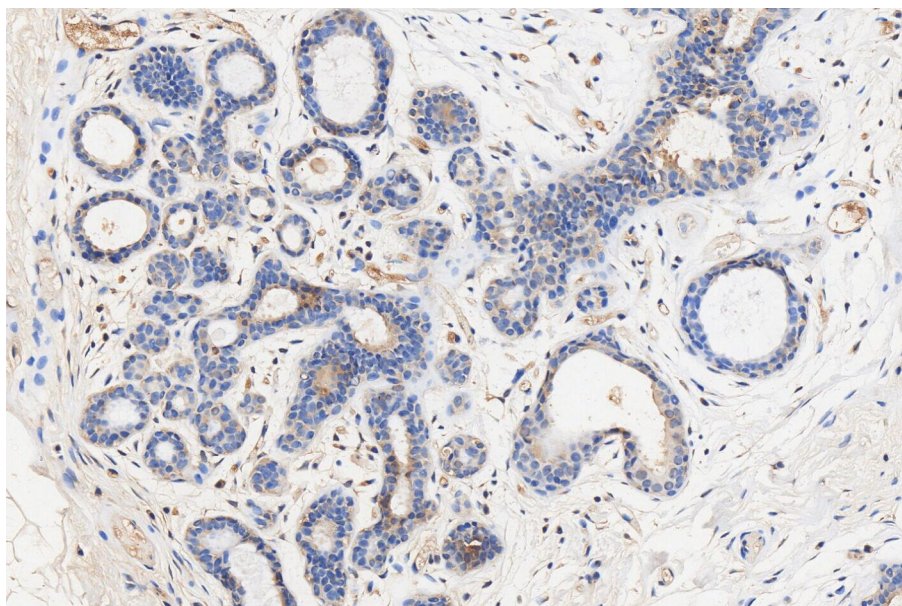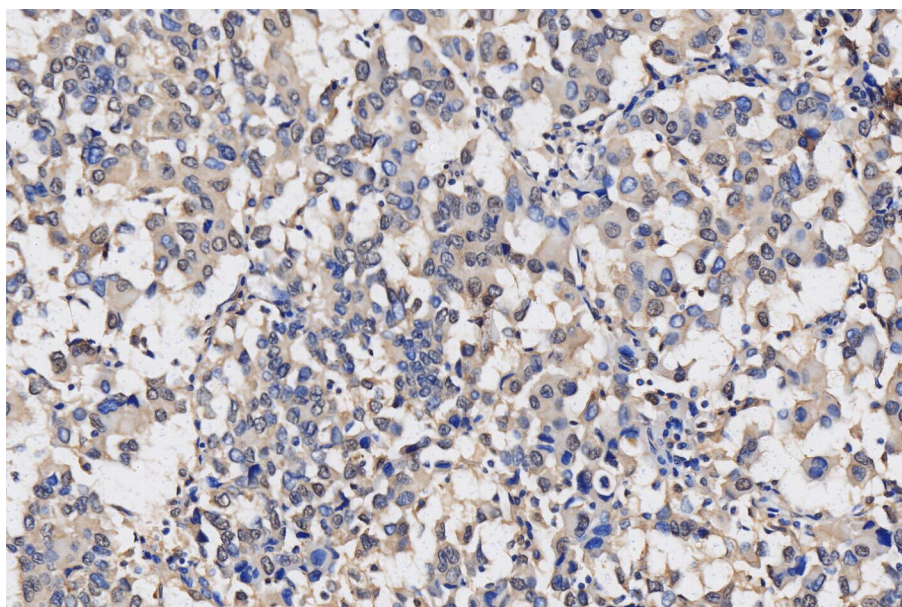

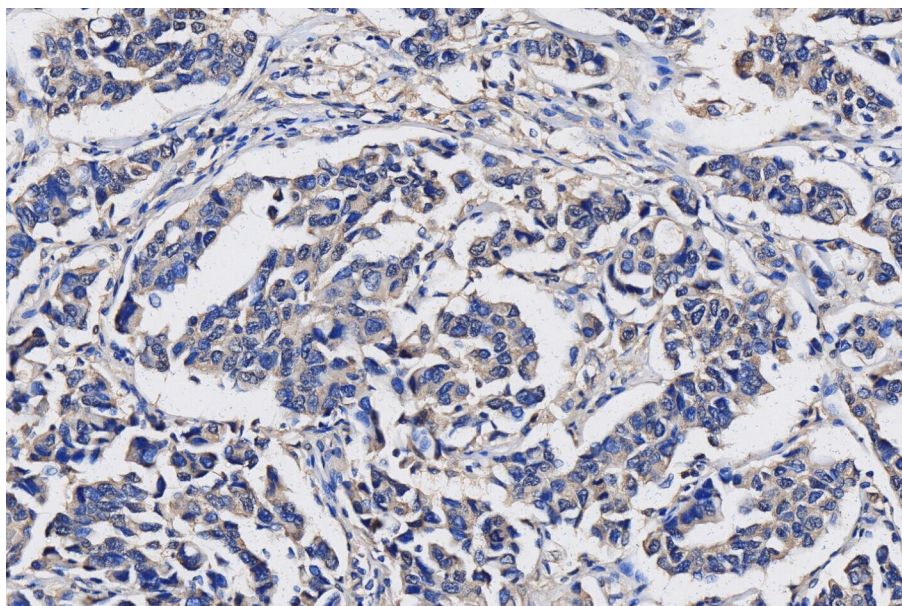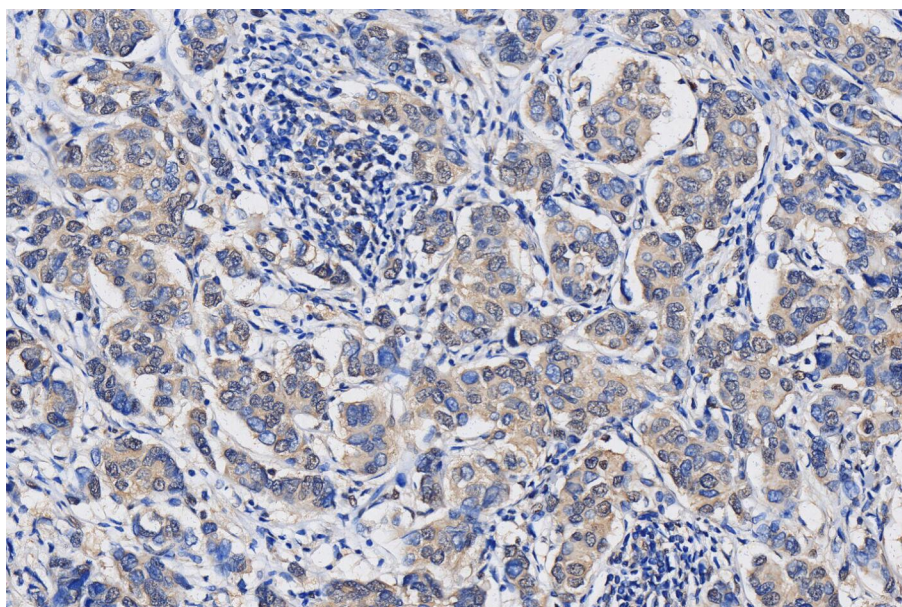

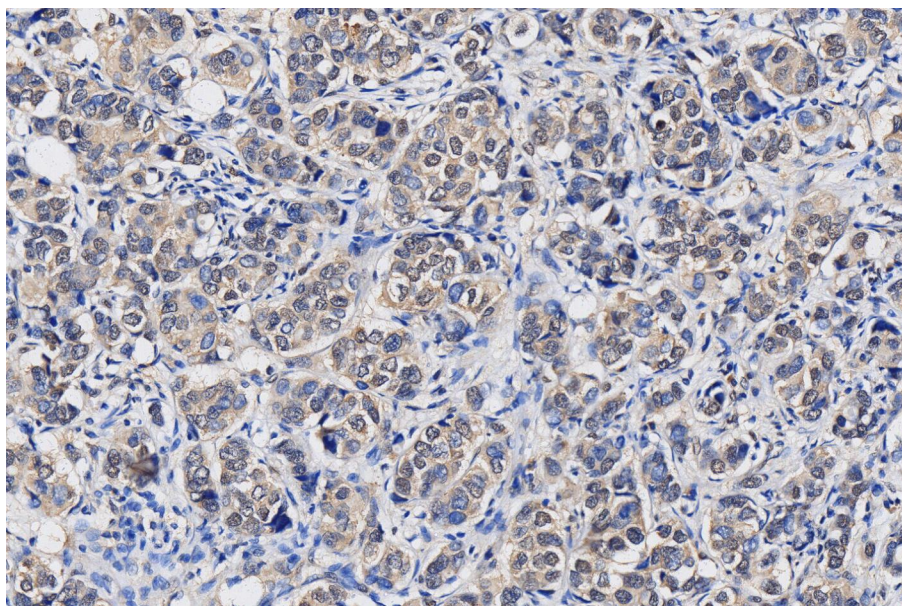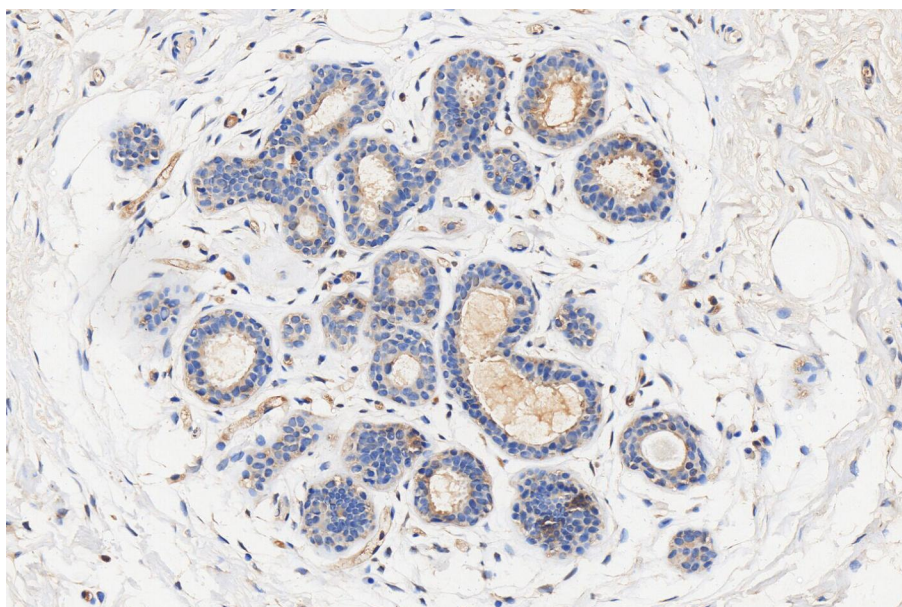

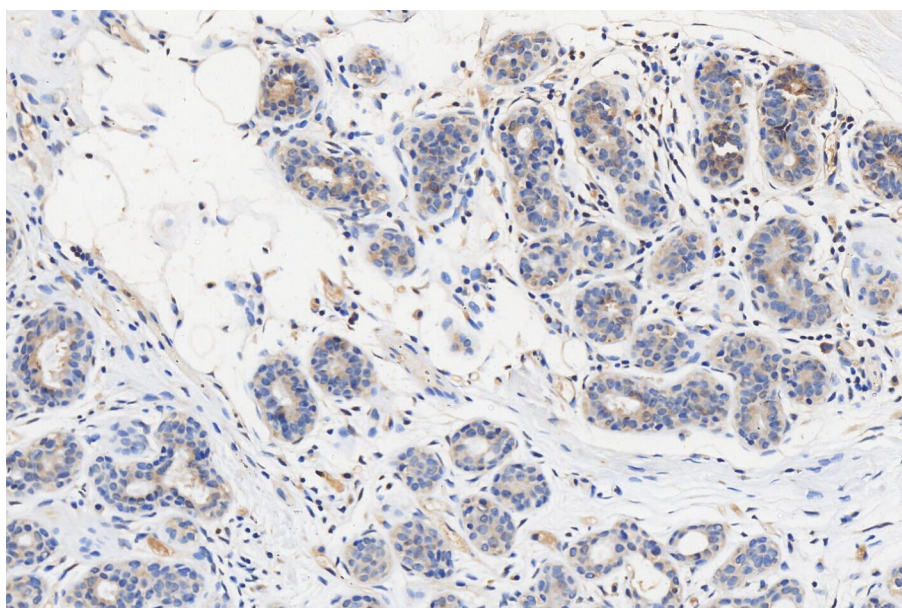

Supplement: Supplementary file 1 [file DataSheet1.zip › Supplementary Materials/Partial Immunohistochemistry Images.pdf]
