# Supplementary material for: Multi-omics analysis of ST3GAL4-mediated lacto/neolacto glycosphingolipid metabolism reveals immune evasion and poor prognosis in TNBC
Source: Front Immunol. 2026 Apr 22;17:1760560. doi: 10.3389/fimmu.2026.1760560 (PMC13143995; doi:10.3389/fimmu.2026.1760560)
Supplement: Supplementary file 2 [file Table1.docx]

**Supplementary Tabale 1** Univariate and multivariate analysis results of TCGA cohort.

| **Characteristics** | **HR(95% CI) Univariate analysis** | **P value** | **HR(95% CI) Multivariate analysis** | **P value** |
| --- | --- | --- | --- | --- |
| **Age** |  |  |  |  |
| <60 | Reference |  | Reference |  |
| >=60 | 1.42 (1.21-1.53) | <0.01 | 1.31 (1.16-1.42) | <0.01 |
| **TNM stage** |  |  |  |  |
| I | Reference |  | Reference |  |
| II | 1.25 (1.11-1.38) | <0.01 | 1.13 (0.92-1.34) | 0.36 |
| III | 1.31 (1.20-1.51) | <0.01 | 1.28 (1.15-1.47) | <0.01 |
| **ST3GAL4** |  |  |  |  |
| Low | Reference |  | Reference |  |
| High | 1.42 (1.31-1.59) | <0.01 | 1.30 (1.12-1.51) | <0.01 |

**Supplementary Tabale 2** Baseline data table of immunohistochemistry cohort patients

| **Clinical and Pathological Data of Each Patient** | |
| --- | --- |
| **Characteristic** | **Value** |
| **cancer type** | TNBC |
| **Age> 60, n (%)** | 73 (0.73) |
| **Ki-67 (>50%), n (%)** | 64 (0.64) |
| **T stage (T1,T2), n (%)** | 37 (0.37) |
|  | 14 (0.140 |
| **N1 stage, n (%)** | 12 (0.12) |
| **N2 stage, n (%)** | 32 (0.32) |
| **N2 stage, n (%)** | 42 (0.42) |
| **Histological grading, (G2)** | 64 (0.64) |

**Supplementary Tabale 3** Univariate and multivariate analysis results of immunohistochemistry cohort.

| **Characteristics** | **HR(95% CI) Univariate analysis** | **P value** | **HR(95% CI) Multivariate analysis** | **P value** |
| --- | --- | --- | --- | --- |
| **Age** |  |  |  |  |
| <60 | Reference |  | Reference |  |
| >=60 | 1.34 (1.12-1.43) | 0.02 | 1.23 (1.08-1.29) | 0.04 |
| **Ki-67** |  |  |  |  |
| <50 | Reference |  | Reference |  |
| >=50 | 1.45 (1.38-1.59) | <0.01 | 1.38 (1.24-1.47) | <0.01 |
| **T stage** |  |  |  |  |
| T1 | Reference |  | Reference |  |
| T2 | 1.18 (1.94-1.34) | <0.01 | 1.04 (0.78-1.27) | 0.86 |
| T3 | 1.21 (1.11-137) | 0.03 | 1.08 (0.94-1.26) | 0.63 |
| **N stage** |  |  |  |  |
| N1 | Reference |  | Reference |  |
| N2 | 1.16 (1.04-1.27) | 0.04 | 1.14 (1.03-1.25) | 0.03 |
| N3 | 1.25 (1.18-1.41) | <0.01 | 1.19 (1.09-1.25) | 0.01 |
| **Histological grading** |  |  |  |  |
| G2 | Reference |  | Reference |  |
| G3 | 0.75 (0.64-0.83) | <0.01 | 0.73 (0.55-0.89) | <0.01 |
| **ST3GAL4** |  |  |  |  |
| Low | Reference |  | Reference |  |
| High | 1.37 (1.28-1.43) | <0.01 | 1.26 (1.15-1.38) | <0.01 |


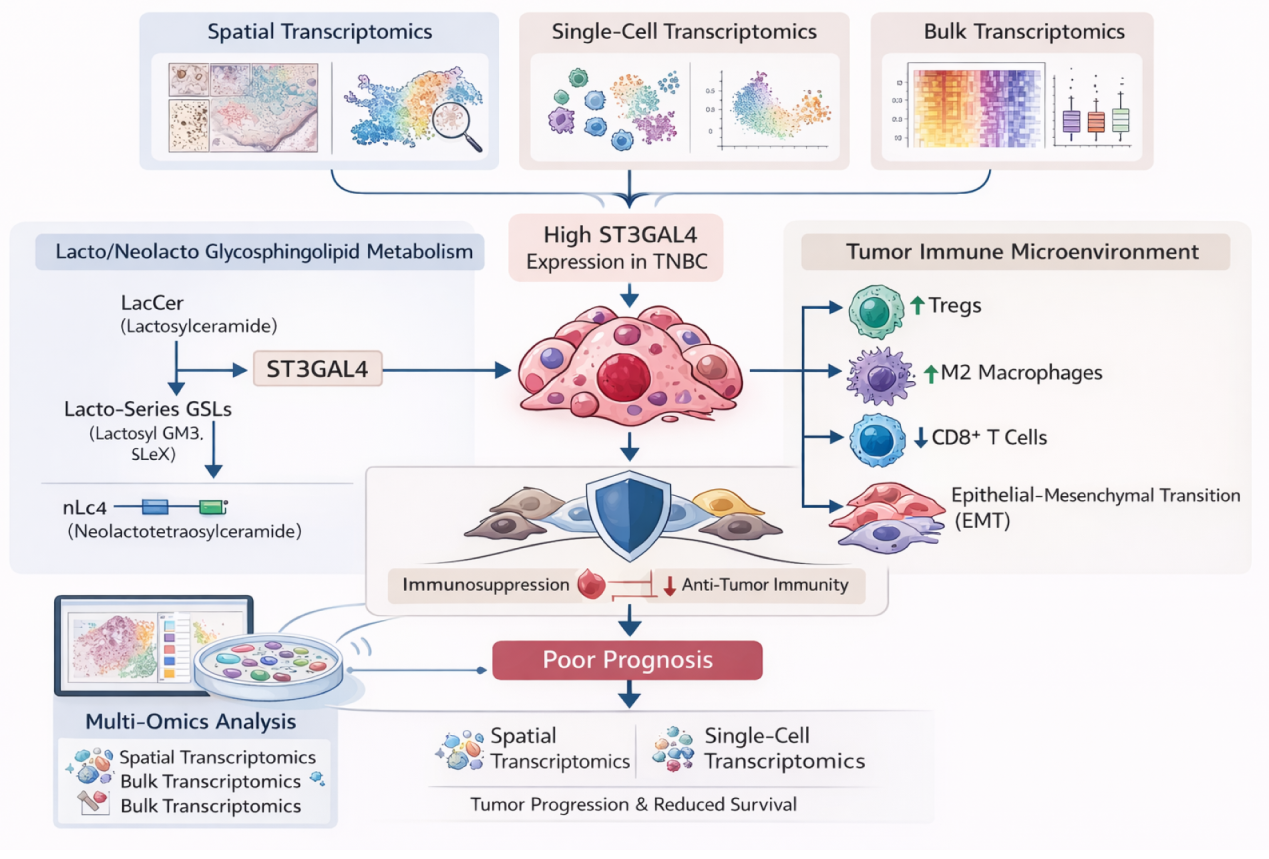


**Supplementary Figure 1** Multi-Omics analysis of ST3GAL4 in TNBC.


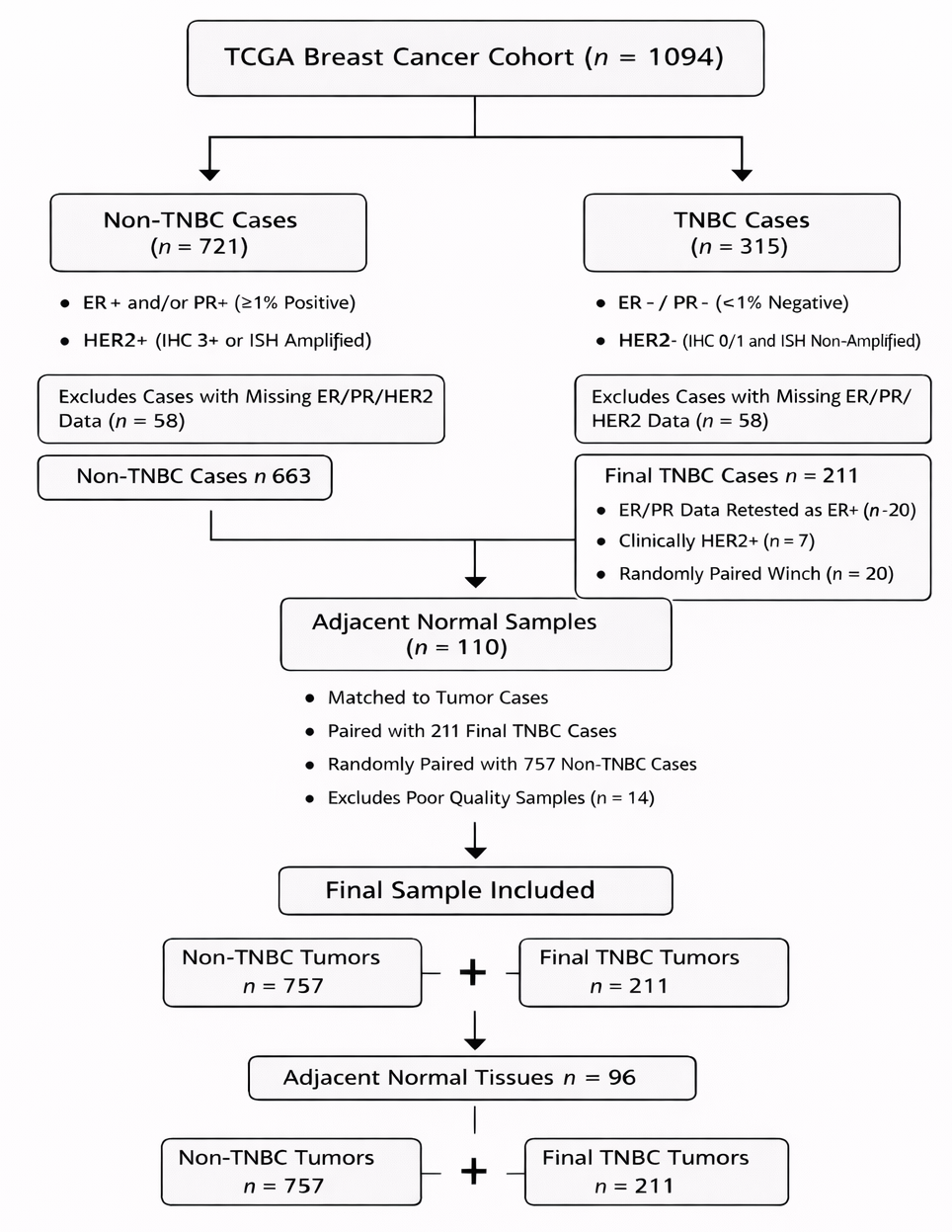


**Supplementary Figure 2** Flowchart of sample selection and exclusion process for the TCGA Breast Cancer cohort.


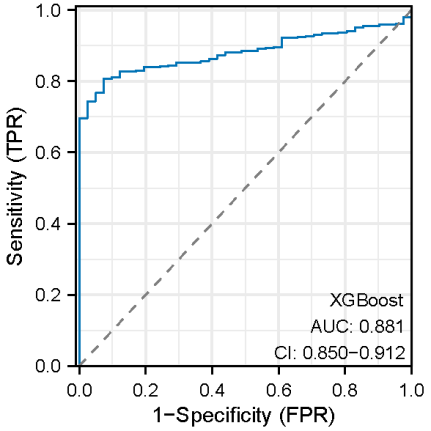


**Supplementary Figure 3** ROC curve analysis of model performance.

**Supplementary Figure 4** The analysis results of ST3GAL4 in pan-cancer.


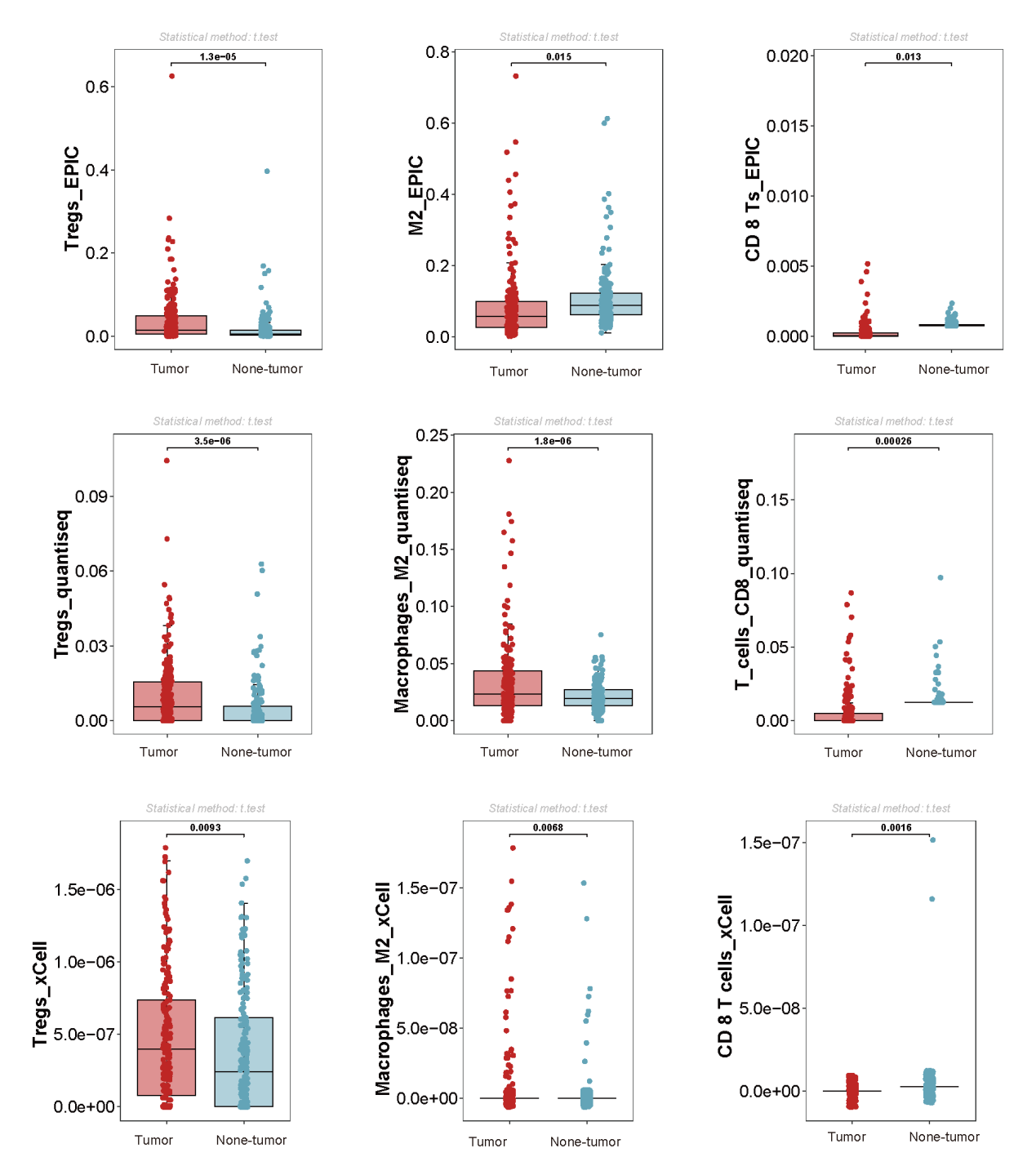


**Supplementary Figure 5** Multiple immune infiltration analysis methods.


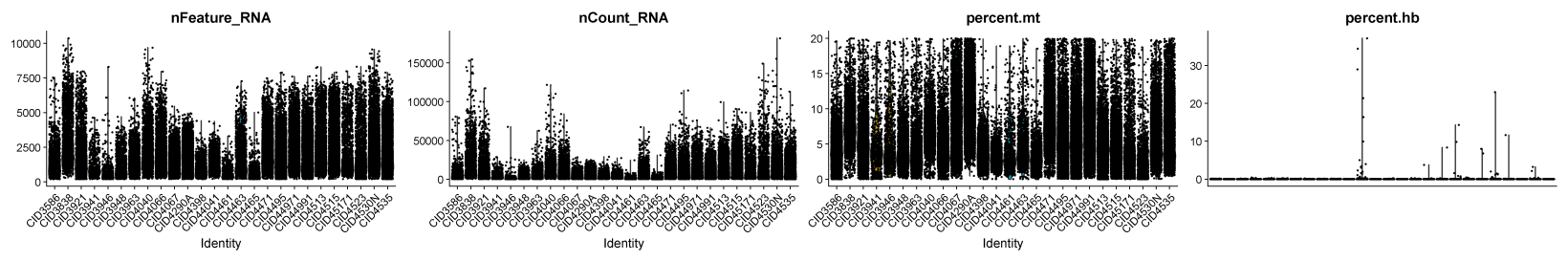


**Supplementary Figure 6 Single-cell quality control criteria.**


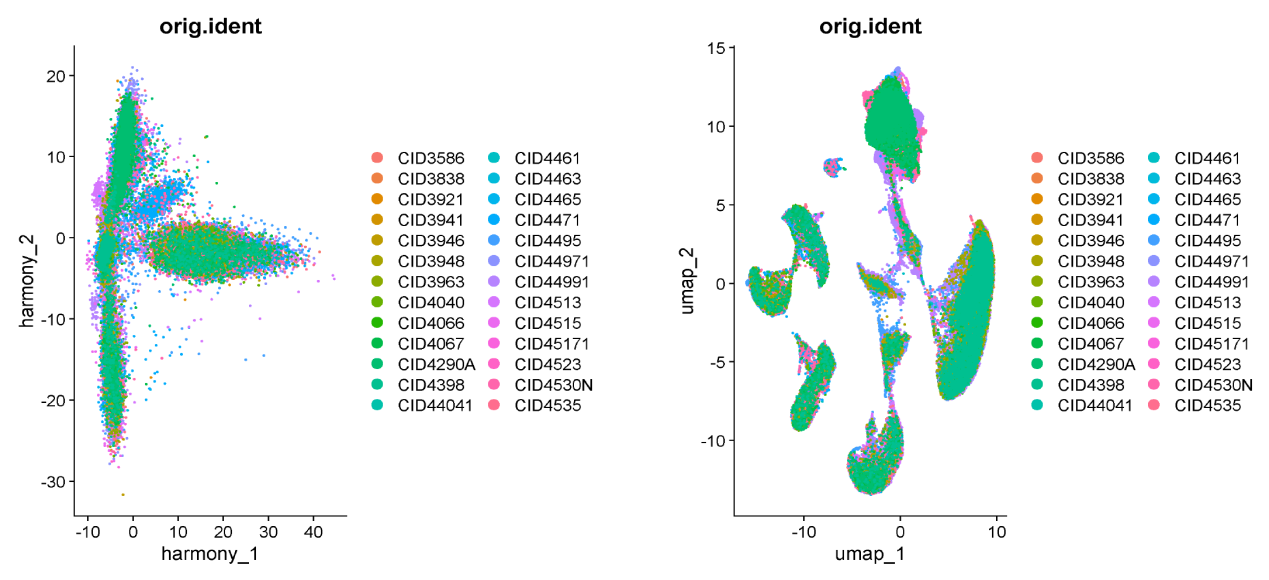


**Supplementary Figure 7** Batch effect correction using harmony on scRNA-seq data


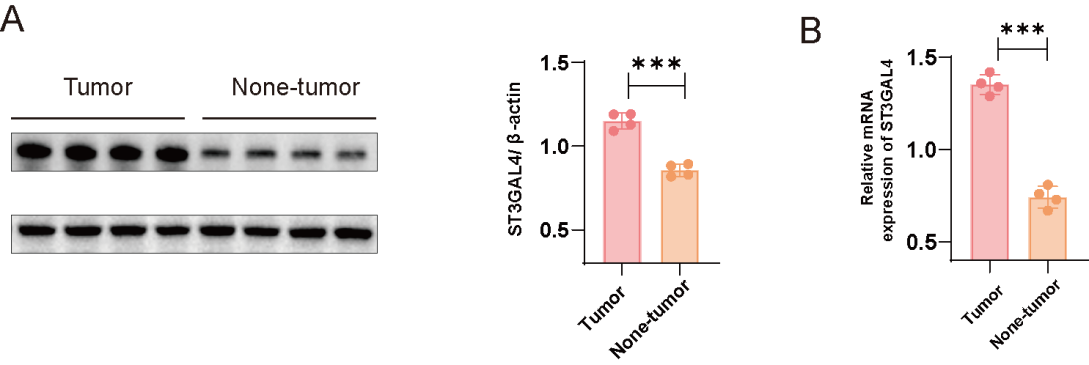


**Supplementary Figure 8** Four cases of TNBC patients were enrolled to assess the expression differences of ST3GAL4 protein and mRNA between tumor and adjacent non-tumor tissues.
